# Co-Pyrolysis of Large-Scale Agricultural Waste as a Mixture of Digestate and Plant Residues: Thermogravimetric Decomposition, Kinetic and Thermodynamic Study, Characterization of Pyrolysis Products

**DOI:** 10.3390/molecules31213863

**Published:** 2026-10-26

**Authors:** Svetlana Timofeeva, Julia Karaeva, Svetlana Islamova, Olga Sidorkina, Ayrat Khamatgalimov

**Affiliations:** 1 Institute of Power Engineering and Advanced Technologies, FRC Kazan Scientific Center, Russian Academy of Sciences, 420111 Kazan, Russia; julieenergy@list.ru (J.K.); isvetulia@mail.ru (S.I.); leylik.sidorkina@mail.ru (O.S.); 2 A.E. Arbuzov Institute of Organic and Physical Chemistry, FRC Kazan Scientific Center, Russian Academy of Sciences, 420029 Kazan, Russia; khamatgalimov@gmail.com

**Keywords:** thermochemical processing, digestate, biomass, co-pyrolysis, utilization, energy

## Abstract

With the continuous increase in agricultural waste, its proper management has become a significant environmental and resource management challenge. This study analyzed the co-pyrolysis of digestate (D) from chicken manure biogas residues and sunflower husks (SH). The weight loss, kinetics during decomposition, and thermodynamic parameters of thermochemical conversion were characterized using a synchronous thermal analyzer. The experiments were conducted at heating rates of 5, 10, 20, and 30 °C/min. The average activation energies for the pyrolysis of D90&SH10, D50&SH50, and D10&SH90 mixtures were obtained using the Kissinger-Akahira-Sunose method and were 274.95 kJ/mol, 239.37 kJ/mol, and 245.09 kJ/mol, respectively. The average activation energies for the pyrolysis of D90&SH10, D50&SH50, and D10&SH90 mixtures were obtained using the Ozawa-Flynn-Wall method and were 367.32 kJ/mol, 321.23 kJ/mol, and 328.52 kJ/mol, respectively. The average activation energies for the pyrolysis of D90&SH10, D50&SH50, and D10&SH90 mixtures were obtained using the Friedman method and were 445.87 kJ/mol, 395.99 kJ/mol, and 358.29 kJ/mol, respectively. The results of thermodynamic analysis confirm favorable conditions for shifting co-pyrolysis reactions towards the formation of stable products; therefore, the process can be carried out in an energy-efficient method. The material balance of thermochemical conversion indicates the potential for producing solid and liquid materials. The results of this study provide valuable information on co-pyrolysis, which contributes to its implementation as an efficient and environmentally friendly waste management strategy.

## 1. Introduction

The management of bulk waste in the agricultural sector is necessary due to its significant contribution to greenhouse gas emissions, water pollution, and ongoing environmental odor problems [1]. According to estimates by the Food and Agriculture Organization of the United Nations (FAO), global livestock production currently accounts for 15% of total anthropogenic greenhouse gas emissions (equivalent to 7.1 gigatons of CO_2_-eq per year). Rational resource use plays a major role in agriculture. Recycling waste into secondary raw materials is of particular interest, as it eliminates or reduces negative environmental impacts. Modern waste recycling technologies also allow for the extraction of valuable components from recycled raw materials and the production of high-value-added products. Recycling agricultural waste also contributes to the development of a circular economy, or economic development in a circular fashion. It should be noted that poultry farming is a major sector of animal husbandry and also a source of enormous waste generation. Chicken manure is a toxic material that causes serious environmental pollution due to its content of excess nitrogen, phosphorus, heavy metals, and pathogens [2]. Over the past few decades, the volume of waste from poultry farms worldwide has increased dramatically due to the growth in meat and egg consumption [3].

The most common methods of poultry waste disposal are landfilling and incineration [4,5], but they cause significant harm to the environment [6]. However, other processing methods are also known, for example: biological (anaerobic digestion) [7,8] and thermochemical (pyrolysis) [9,10]. Previously, these technologies were often opposed to each other, but currently, the combination of pyrolysis and anaerobic digestion processes is attracting increasing attention [11]. The integration of biological and thermochemical conversion technologies contributes to the expansion of the range of processed raw materials, and also allows for the transition to a circular economy.

The digestate has limitations in terms of quantity and timing of application to fields, due to both climatic conditions and the need to avoid nitrogen and phosphorus leaching into water bodies and groundwater. High humidity (over 90%) creates difficulties during storage and transportation [5,7,8]. The presence of pathogenic microorganisms and high nitrogen content in chicken manure digestate also poses an environmental threat.

Biogas production processes are characterized by incomplete conversion of feedstock, making digestate a promising material for the production of new products using other processing methods. Waste management from biogas plants is an important task, and thermochemical conversion of digestate to produce new value-added products represents a promising solution. Combining two technologies enables comprehensive, deep biothermochemical processing and a full cycle of organic waste recycling. Such a technology could not only be energy-independent but also produce high-added-value products, such as biochar and pyrolysis liquid, solid composite materials, and valuable fertilizers [1,3].

The relevance of solving this problem is due to the insufficient study of the fundamental principles and mechanisms of individual stages of the biothermochemical conversion of organic matter, as well as the lack of data in the scientific and technical literature on combining biological (anaerobic digestion) and thermochemical (pyrolysis) processes within a single organic waste disposal technology.

This study will enable the implementation of a “cascade” approach, in which the products of one process become raw materials for the next, i.e., it is also aimed at achieving the goal of “waste-free production”, which allows for the transformation of most of the organic matter present in the waste [12]. Furthermore, digestate cannot always be applied to soil on an industrial scale due to climatic conditions, the risk of environmental pollution, strict legal regulations, logistical constraints, and high costs [13]. Therefore, the thermochemical conversion of digestate or sludge from the biological conversion of poultry waste is of particular interest. It should be noted that such studies are currently lacking in published literature.

Another aspect of thermochemical processing within the combined technology is the possibility of drying the digestate. For this purpose, it is advisable to use the energy obtained from the combustion of pyrolysis gas. Pyrolysis is an environmentally sustainable technology for processing chicken manure [14]; however, improving the quality of the resulting products requires the addition of other feedstocks, such as those of plant origin. Pyrolysis of poultry waste alone, i.e., without the addition of biomass or other waste, has also not gained widespread acceptance due to the resulting products having unsatisfactory characteristics [10,11]. Chicken manure, when used as a standalone feedstock for pyrolysis, has certain drawbacks: high moisture content, high ash content, and an excess of nitrogen. Adding carbon-rich plant-based feedstock optimizes the C/N (carbon-to-nitrogen) ratio and stabilizes the pyrolysis process.

Co-pyrolysis of chicken manure with lignocellulosic materials significantly improves the treatment process. This method effectively converts toxic raw materials into valuable products, such as a solid product, pyrolysis gas, and biofuel. Chicken manure is high in nitrogen and mineral components, and often contains alkali metals [11,13,14]. The addition of plant biomass increases the carbon content and improves the carbon-to-nitrogen ratio. The addition of lignocellulosic material also increases the carbon content of the solid product, improving its quality. Furthermore, the solid products from the pyrolysis of pure chicken manure are highly toxic, containing elevated salt and heavy metal levels.

Biochar obtained from poultry manure consistently demonstrated the highest ecotoxicity regardless of the pyrolysis temperature, highlighting the critical role of feedstock selection [15]. In this regard, the addition of biomass makes the resulting solid product safe for use. Co-pyrolysis of chicken manure with tree bark was investigated to mitigate toxic element concentrations of biochar (polycyclic aromatic hydrocarbons, zinc, copper, and chromium content) [16]. To obtain higher quality products during thermochemical conversion, sugarcane bagasse was added to poultry manure [10]. A simple technology for the co-pyrolysis of chicken manure and natural calcium bentonite to obtain a slow-release fertilizer based on biochar is known [17]. It was found that co-pyrolysis of chicken manure with phosphates and magnesium also allows one to obtain a slow-release fertilizer suitable for tropical soils [18]. To enable the use of poultry manure as a source of raw material for energy production, it was co-pyrolyzed with biomass: corn stalk, wheat straw, and pine sawdust [19]. Thus, co-pyrolysis technologies, by combining several types of raw materials, improve the characteristics of the resulting products and contribute to waste reduction [20]. This study proposes using sunflower husks for mixing with wastewater, which is feasible given that this lignocellulosic raw material is a high-volume agricultural waste. From an economic perspective, if the use of this agro-industrial waste reduces disposal costs and allows for the use of cheaper feedstocks in thermal conversion processes, then the reduced waste disposal volume and the possibility of partially replacing it with fossil fuels will contribute to more sustainable development.

According to the FAO (2000–2022), “the global cultivated area of sunflower exceeds 404,686 km^2^, making it the third most important oilseed crop after soybean and rapeseed” [21]. World sunflower seed production was estimated to be 58 million metric tons in 2022, and the sunflower husk generated was about 14 million cubic meters [22]. At oil extraction plants, finding new methods for utilizing sunflower husks is a major challenge. Currently, they are most often used to produce fuel pellets [23]. Storing sunflower husks in landfills results in greenhouse gas emissions of approximately 433 kg CO_2_ eq/t [24]. Furthermore, using this feedstock for thermochemical conversion is economically feasible, as it is produced in large volumes and is relatively unaffected by the seasons, unlike other types of biomass.

Several factors prompted us to conduct the study described in this article. A literature review showed that there are knowledge gaps in many of the aforementioned areas. For example, there are no studies on the pyrolysis of high-ash digestates, including digestate from chicken manure with sunflower husk. Data on kinetic and thermodynamic parameters of thermochemical conversion are also lacking. In addition, there is limited information about the effect of sunflower husk addition to high-ash digestate on the composition of pyrolysis products. To address the aforementioned knowledge gaps, this study examined the thermogravimetric behavior of three mixtures of digestates obtained as a result of anaerobic digestion of chicken manure with sunflower husk. Thermogravimetric analysis (TGA) coupled with Fourier transform infrared analysis (FTIR). Kinetic analysis was carried out using three isoconversion methods: Friedman, Kissinger-Akahira-Sunose (KAS), and Ozawa-Flynn-Wall (OFW). The resulting pyrolysis chars were characterized using several analytical methods, which included proximate analysis, X-ray fluorescence analysis, FTIR analysis. The pyrolysis liquid was characterized using GC-MS.

This work introduces several innovations. As far as the authors know, the co-pyrolysis kinetics of digestate (D) and lignocellulosic raw material—sunflower husk (SH)—were studied for the first time. Thermodynamic parameters such as Δ*H*, ΔG, and ΔS were also determined, which is not found in the literature for this type of digestate. The data obtained in experiments on pyrolysis products represent a significant novelty, as they provide valuable information about the possible applications of the resulting liquid and solid products.

## 2. Results

### 2.1. Results of Proximate and Ultimate Analyses

The results of the proximate and ultimate analyses of the studied samples are presented in Table 1.

The samples had the following names and compositions:

D90&SH10: a mixture of 90 wt% digestate and 10 wt% sunflower husks.

D50&SH50: a mixture of 50 wt% digestate and 50 wt% sunflower husks.

D10&SH90: a mixture of 10 wt% digestate and 90 wt% sunflower husks.

The ultimate analysis is consistent with the data for lignocellulosic materials from plant biomass and animal waste [25,26]. The results of the ultimate analysis show that D10&SH90 contains more carbon, hydrogen, and oxygen compared to D50&SH50 and D90&SH10 samples. Higher carbon and hydrogen content contributes to an increase in the energy density of the feedstock and promotes the release of CO and CO_2_ during pyrolysis through decarbonylation and decarboxylation reactions [27].

The studied samples have been characterized by low moisture content, which contributes to an increase in thermal efficiency during thermochemical conversion. High ash content values correspond to samples D90&SH10, D50&SH50, where the main component of the mixture is D, which is characterized by a large amount of mineral substances in the source material. For sample D10&SH90, the ash content is 9.2 wt%, which is due to the low content of mineral substances in sunflower husks. The amount of volatiles is in the range from 47.1 to 73.1 wt%, which corresponds to the average values for lignocellulosic feedstock. Sample D90&SH10 has a low yield of volatiles, which causes its low reactivity, indicating a slower stage of volatile release during thermochemical conversion. Sample D10&SH90 has high reactivity. HHV of the studied samples has average values, which is consistent with the literature data [28,29].

### 2.2. Thermal Analysis Results

#### 2.2.1. TGA Results

**Figure 1:**
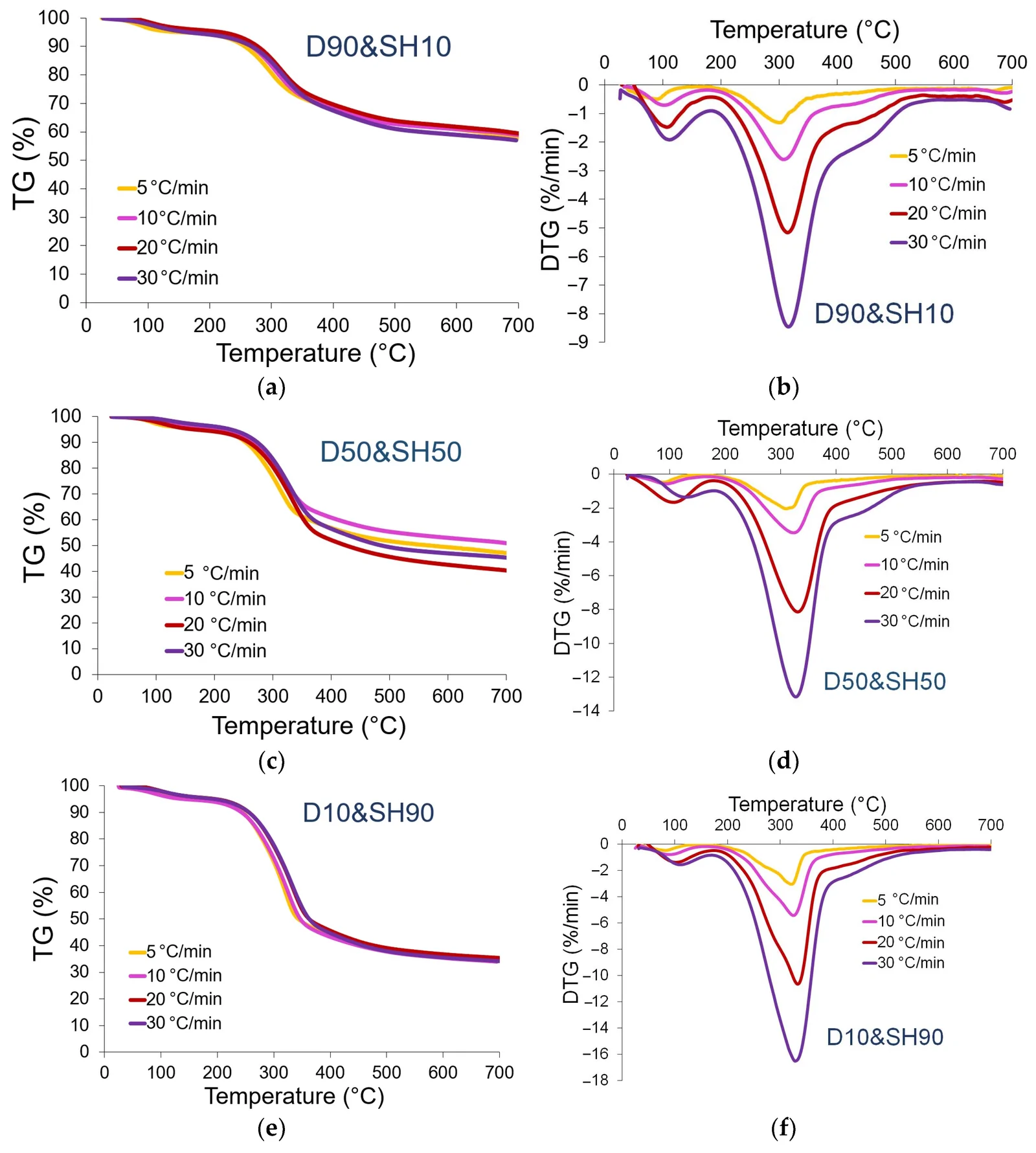
TGA Results: TG curves of D90&SH10 (**a**), DTG curves of D90&SH10 (**b**), TG curves of D50&SH50 (**c**), DTG curves of D50&SH50 (**d**), TG curves of D10&SH90 (**e**), DTG curves of D10&SH90 (**f**).

As a result of the TGA co-pyrolysis of the samples, thermogravimetric curves (TG) and derivative thermogravimetric curves (DTG) were obtained at heating rates of 5, 10, 20, and 30 °C/min. The nature of these changes is presented in Figure 1.

As a result of the studies, the main temperature ranges of individual stages characteristic of the thermochemical conversion of lignocellulosic feedstock and animal biomass were determined. Thermogravimetric data for the initial samples D and SH were analyzed, and the results are presented in [30]. The thermal decomposition process of the studied samples consists of the stages of dehydration, devolatilization, and degradation of char and minerals (Table 2).

The mass loss in the TG curves up to 190 °C for all samples is associated with the removal of residual moisture and adsorbed water from the surface and pores of the biomass, which corresponds to the drying process [31,32]. The second stage of co-pyrolysis is characterized by the greatest mass loss and is associated with the release of volatile substances from the mixture of D and SH as a result of the thermal decomposition of cellulose and hemicellulose, as well as carbohydrates and proteins, which is confirmed by literature data for similar mixtures [29,33,34]. Cellulosic substances (carbohydrates and polysaccharides) begin to decompose when heated above 250 °C, glycosidic bonds are broken and a complex mixture of gases (pyrolysis gas) is formed. Cellulose is the main component of SH, its content ranges from 50% to 60% [35]. Hemicellulose is also present in significant quantities, about 20–30% [31]. Component D is characterized by the decomposition of organic compounds in the material, such as carbohydrates and proteins, with the formation of volatile compounds [36,37]. At this stage of active decomposition, peaks are observed on the DTG curves, characterizing the maximum decomposition rate of the samples during pyrolysis. The maximum rate of mass loss is typical for a heating rate of 30 °C/min. At temperatures of 320 °C and above, the mass loss for all samples slows down. At this temperature, complex thermal decomposition of lignin begins to occur. Lignin decomposition occurs slowly over the entire temperature range up to 900 °C [38]. The thermal decomposition of lignin begins in the 200–250 °C range. The process proceeds most intensely at temperatures between 300 and 450 °C [39]. The DTG curves also show a decrease in the mass loss rate.

In the third stage, at temperatures above 550–600 °C, the mass loss process stabilizes, and the reaction enters the stage of secondary carbonization and mineral decomposition [36]. For the biomass component (SH), this stage is characterized by the slow degradation of the highly condensed aromatic lignin matrix and the formation of a stable carbonaceous residue structure. For the chicken manure component (D), a key feature of this high-temperature zone is the thermal decomposition of inorganic substances and mineral salts—such as calcium carbonate (CaCO_3_), which typically undergoes calcination at 600–750 °C to form CaO and release secondary CO_2_ [33,36]. At temperatures above 600 °C, the TG curves reach a constant mass, leaving a solid residue composed of fixed carbon (biochar) and a mineral-rich ash fraction.

#### 2.2.2. TG-FTIR Analysis

**Figure 2:**
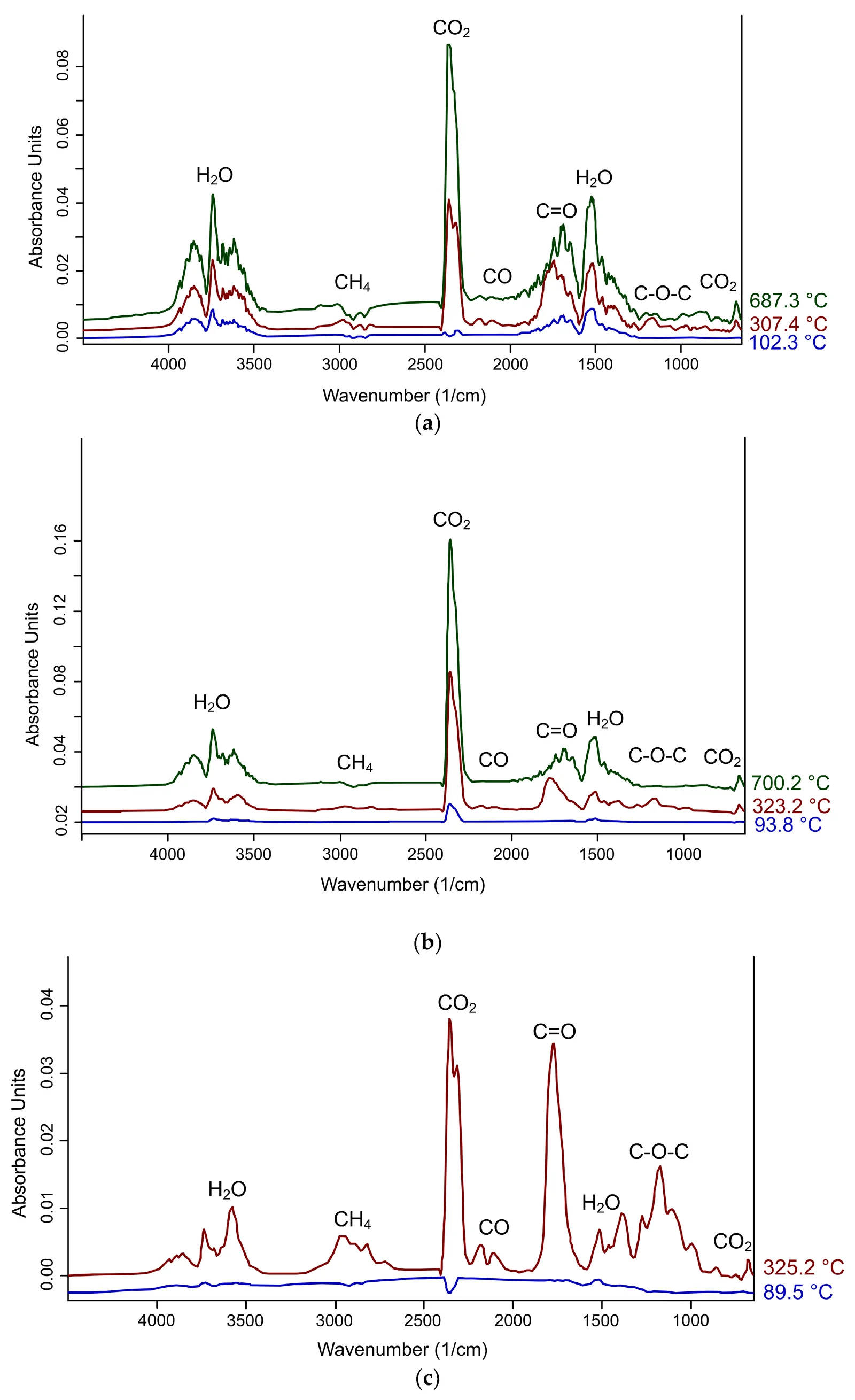
FTIR spectra of gaseous products: D90&SH10 (**a**), D50&SH50 (**b**), and D10&SH90 (**c**).

Signals of gases released during decomposition in a TG analyzer were obtained using an FTIR spectrometer. Gram–Schmidt curves were constructed, reflecting the intensity of the absorption maxima of gaseous substances released at a heating rate of 10 C/min. These conditions correspond to the co-pyrolysis process in the laboratory setup. As a result of the study, the areas of highest gas generation were identified (Figure 2).

Vibrations in the 1250–1600 and 3500–4000 cm^−1^ wavenumber ranges of the spectra are associated with the formation of water vapor resulting from the dehydration and decomposition of oxygen-containing groups. In the wavenumber ranges of 2240–2400 and 700–750 cm^−1^, the detected spectra are a consequence of the vibration of the CO_2_ molecule. Minor bands in the range of 2750–3100 cm^−1^ are associated with vibrations of methyl and methylene groups [40]. The absence of pronounced bands at 3050 cm^−1^ and weakly noticeable bands in the range of 2020–2220 cm^−1^ indicate vibrations of CH_4_ and CO. Spectral bands in the range of 900–1250 cm^−1^ indicate the presence of an ether (C–O–C). These results confirm the findings of previous FTIR studies on manure/biomass pyrolysis, which established that C–O–C functional groups are among the primary volatile species released during the initial mass loss stage within the temperature range of 200 to 500 °C [41]. Absorption bands in the ranges 1400–1600 and 1600–1850 cm^−1^ indicate the presence of aromatic hydrocarbons (C=C, =C–H) and carbonyl compounds (C=O), respectively. The observed stretching vibrations indicate the presence of ketones, quinones, and/or carboxyl carbon [42]. The spectra for the D90&SH10 and D50&SH50 samples at temperatures of 687.3 and 700.2 °C characterize the peaks of H_2_O, CO_2_, and volatile carbonyl compounds (acids, aldehydes, ketones). The bands at 1500, 2240–2400 cm^−1^ became more intense, which is clearly visible in Figure 2a,b. For higher pyrolysis temperatures, gas signals were obtained for the D10&SH90 sample.

IR spectra of the gaseous products revealed that peaks of H_2_O and CO_2_ gas release, as well as carbonyl groups, are recorded in samples D90&SH10 and D50&SH50 (at 687.3 and 700.2 °C). The bands at 1500 and 2240–2400 cm^−1^ increase significantly. However, sample D10 and SH90 do not produce strong gas signals at higher pyrolysis temperatures. The multi-stage decomposition profile of the components in the studied samples is similar to that previously observed in other manure-based materials, where thermal decomposition is characterized by stages of drying, degassing, and char formation/carbonization [43].

#### 2.2.3. Interaction Between Co-Pyrolysis of D and SH

**Figure 3:**
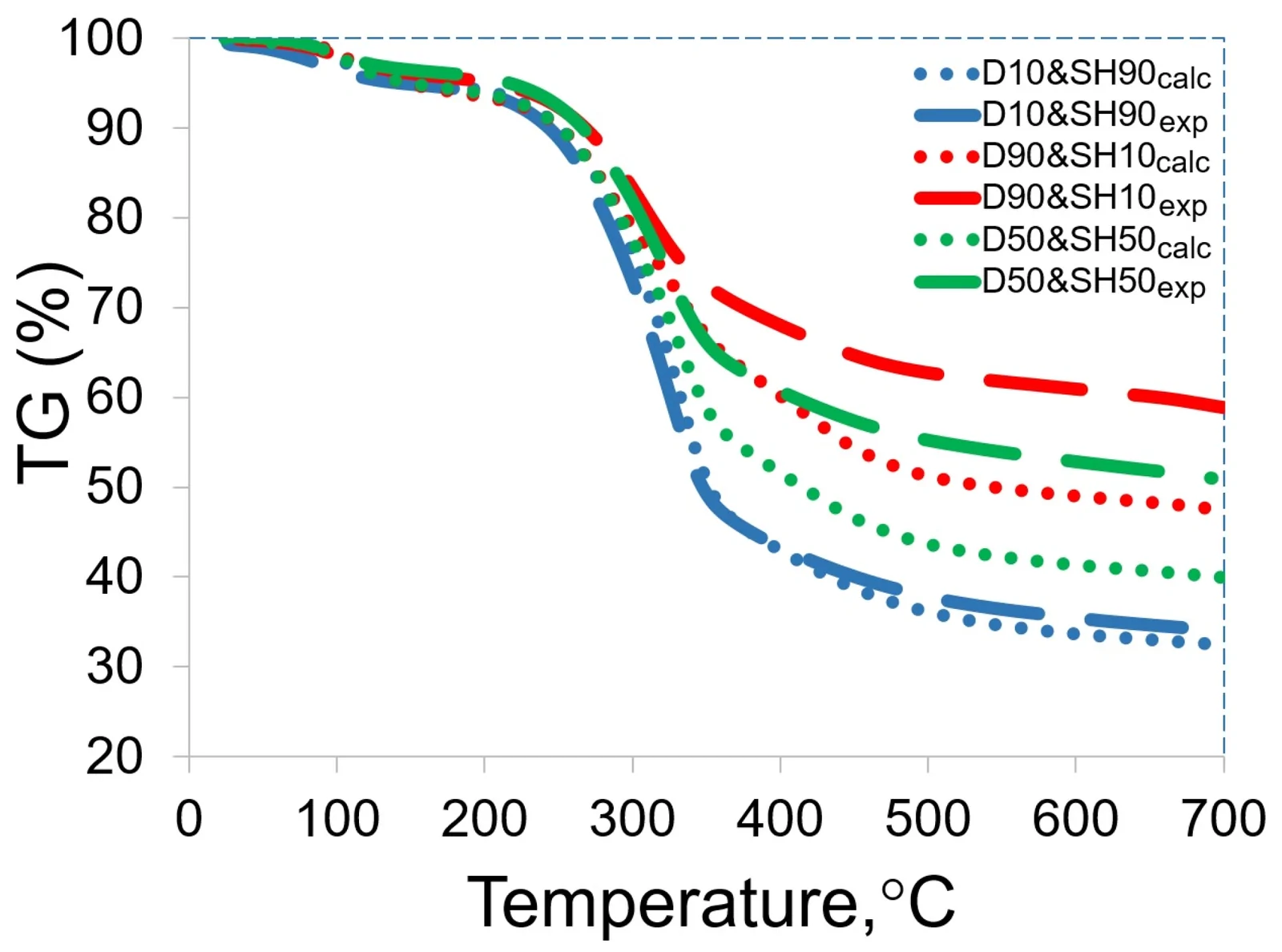
TG curves comparison between the experimental and calculated value.

The interaction between D and SH is shown by the difference in the thermogravimetric curves (Figure 3) at a heating rate of 10 °C/min.

As shown in Figure 3, the experimental TG profiles exhibited delays and were shifted to the right on the graphs. Co-pyrolysis dominated by SH (sample D10&SH90) is characterized by a single region exhibiting a positive synergistic effect. At temperatures up to 370 °C, this effect is attributed to the contribution of hemicellulose and cellulose to chemical transformations during the stage of active volatile release. Chemical decomposition and depolymerization reactions played a dominant role in the co-pyrolysis process within the low-temperature range for the mixture containing chicken manure [44]. Consequently, it is advisable to investigate the properties and structure of solid products obtained from the co-pyrolysis of complex mixtures—such as the D and SH studied here—where the SH content exceeds 50%, a practice also recommended in other studies [17,45].

A slight positive synergistic effect resulting from the addition of SH was observed during the co-pyrolysis of samples D90&SH10 and D50&SH50 in the low-temperature drying zone. However, as the temperature increased, a negative effect was detected throughout the thermal decomposition range.

#### 2.2.4. Kinetic Analysis Results

In this study, the kinetic parameters of the samples were determined using the OFW, KAS, and Friedman model-free methods based on TGA data. The conversion range chosen was *α* = 0.1–0.7, which was due to instability near the end of the reaction, consistent with [46]. For each model-free method, the slope of the straight line was obtained from the linear regression results, and the *E_α_* values were calculated (Table 3).

Changes in the activation energy values depending on the conversion are evident due to differences in the degree of decomposition of the constituent components. Table A1 presents the mean activation energy values along with the standard deviation. The co-pyrolysis mechanism of the samples is quite complex due to their inhomogeneous heterogeneous composition.

**Figure A1:**
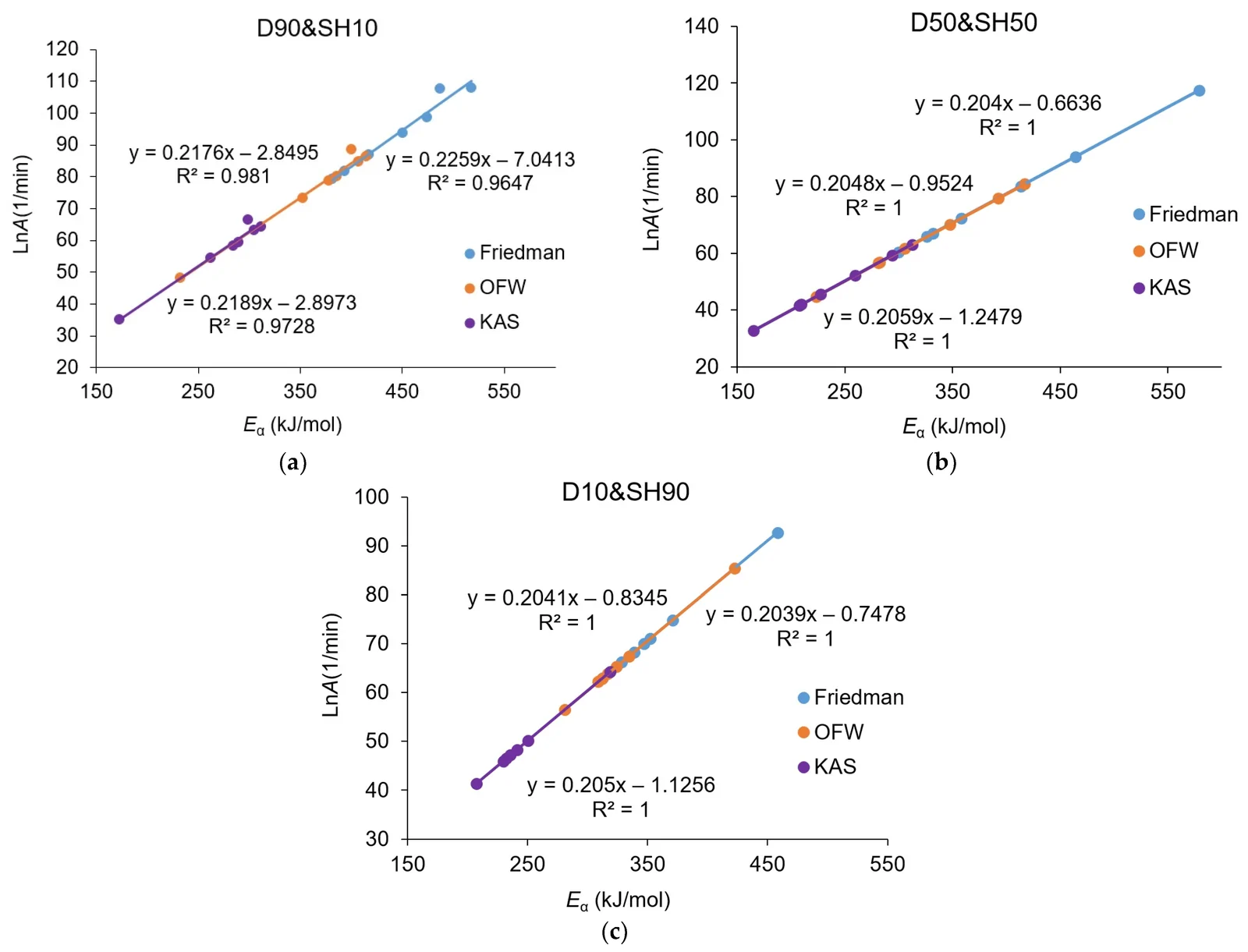
Kinetic compensation effect for samples D90&SH10 (**a**), D50&SH50 (**b**), and D10&SH90 (**c**).

The *E_α_* value shows a measure of the minimum energy required to initiate a chemical reaction and is also a potential measure of reactivity [47]. The average activation energies *E_α_* for the pyrolysis of D90&SH10 were 367.32 kJ/mol (232.77–414.88 kJ/mol), 274.95 kJ/mol (173.14–311.70 kJ/mol), and 445.87 kJ/mol (381.29–517.76 kJ/mol) according to the OFW, KAS, and Friedman methods, respectively. The average activation energies for the pyrolysis of D50&SH50 were 321.23 kJ/mol (223.45–416.89 kJ/mol), 239.37 kJ/mol (164.94–312.41 kJ/mol), and 395.99 kJ/mol (299.66–579.15 kJ/mol) according to the OFW, KAS, and Friedman methods, respectively. The average activation energies for the pyrolysis of D10&SH90 were 328.52 kJ/mol (280.90–422.58 kJ/mol), 245.09 kJ/mol (207.49–318.98 kJ/mol), and 358.29 kJ/mol (312.38–458.32 kJ/mol) according to the OFW, KAS, and Friedman methods, respectively. The obtained pre-exponential factors *A* were elevated (more than 10^10^ 1/min), indicating weak bonds between the activated complexes formed during pyrolysis, which indicates the manifestation of a kinetic compensation effect [48]. To confirm the existence of the kinetic compensation effect, plots of ln A versus *E_α_* were constructed (Figure A1).

**Figure 4:**
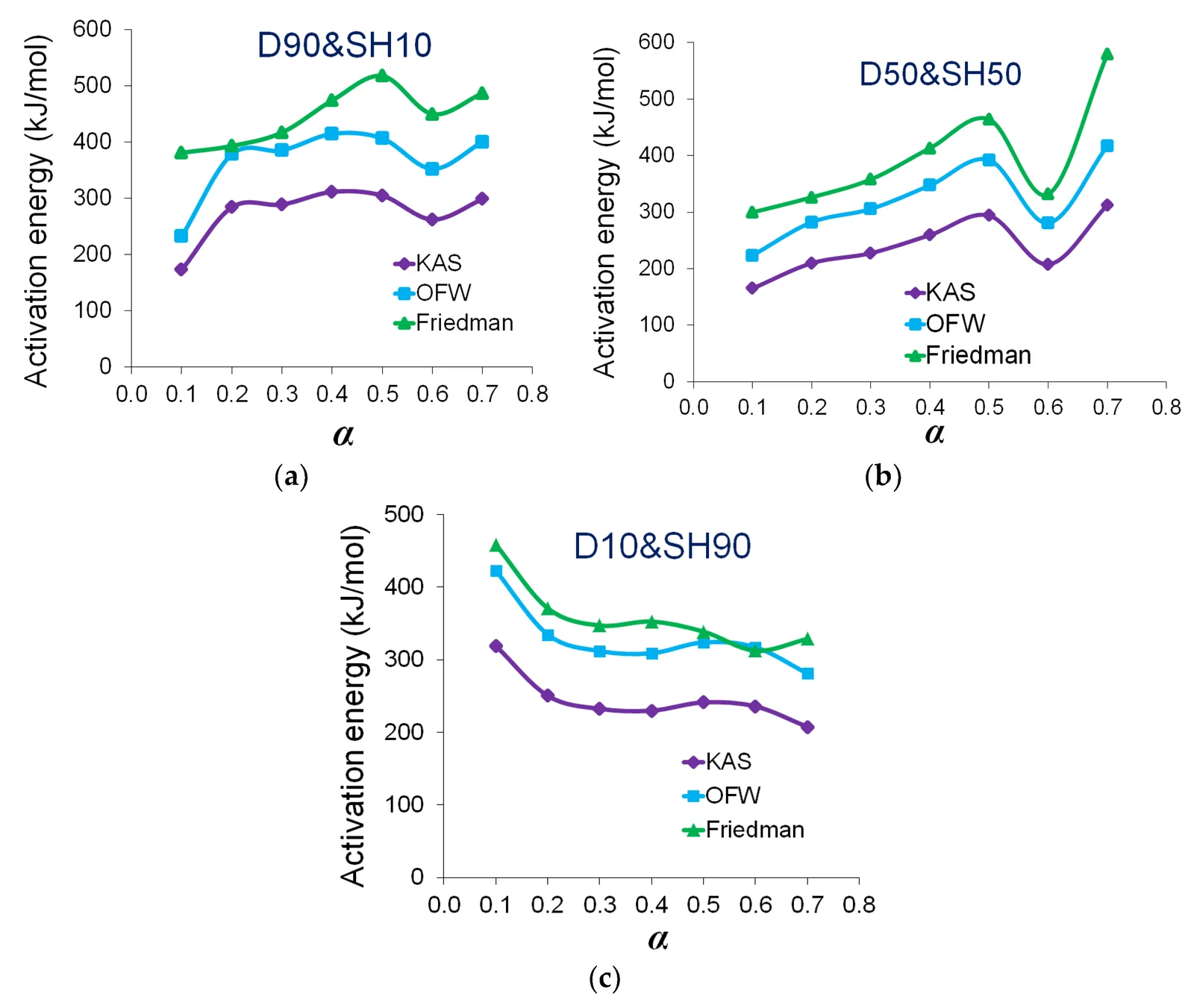
Dependence of activation energy values on the degree of conversion for samples: D90&SH10 (**a**), D50&SH50 (**b**), and D10&SH90 (**c**).

Figure 4 shows the curves of changes in activation energy depending on the conversion. The shape of the *E_α_* curves confirms that the lignocellulosic content of the biomass makes the pyrolysis mechanism quite complex. In the case of D90&SH10, the shape of the OFW and KAS curves is almost identical, reaching a peak at α = 0.4. The Friedman method shows a consistently higher activation energy value for all degrees of conversion, reaching a peak at α = 0.5, after which it decreases again. The final stage at α > 0.6 requires a higher activation energy for lignin decomposition, which is noticeable in all methods.

The increase in activation energy values at the end of the pyrolysis process can be explained by the low volatility and high stability of the lignin structure [49], as well as changes in the pyrolysis mechanism [50,51]. The curves for sample D50&SH50 are similar in nature to those for sample D90&SH10, with the maximum Eα values occurring at the very end of the conversion range at α = 0.7. A higher activation energy indicates that the reactions proceed more slowly. For sample D10&SH90, the curves showed a smooth downward trend with a maximum at α = 0.1 and α = 0.6–0.7.

The two kinetic methods employed (FWO and KAS) yielded similar activation energy values at each conversion level; this was expected, as they are integral isoconversional methods derived from equivalent temperature-integral approximations that typically converge to similar results. The Friedman method yielded a higher average value, a result attributed to the fact that this method utilizes instantaneous reaction rates for a specific degree of conversion [52].

High activation energy values reflect the greater thermal stability of the more condensed and aromatic structures found in the studied samples. Consequently, significantly more energy is required to decompose these structures. Overall, this trend is largely consistent with findings for other manure or poultry litter feedstocks analyzed using various isoconversional methods, as well as with studies on cow manure pyrolysis, where average activation energy (*E_α_*) values obtained via the FWO, KAS, and Friedman methods ranged from 150 to 400 kJ/mol [46,48]. The magnitude of the activation energy values and their dependence on the conversion degree also fall within the broader ranges reported for lignocellulosic and agro-industrial biomass wastes analyzed using the aforementioned kinetic methods [46].

#### 2.2.5. Thermodynamic Analysis Results

**Figure 5:**
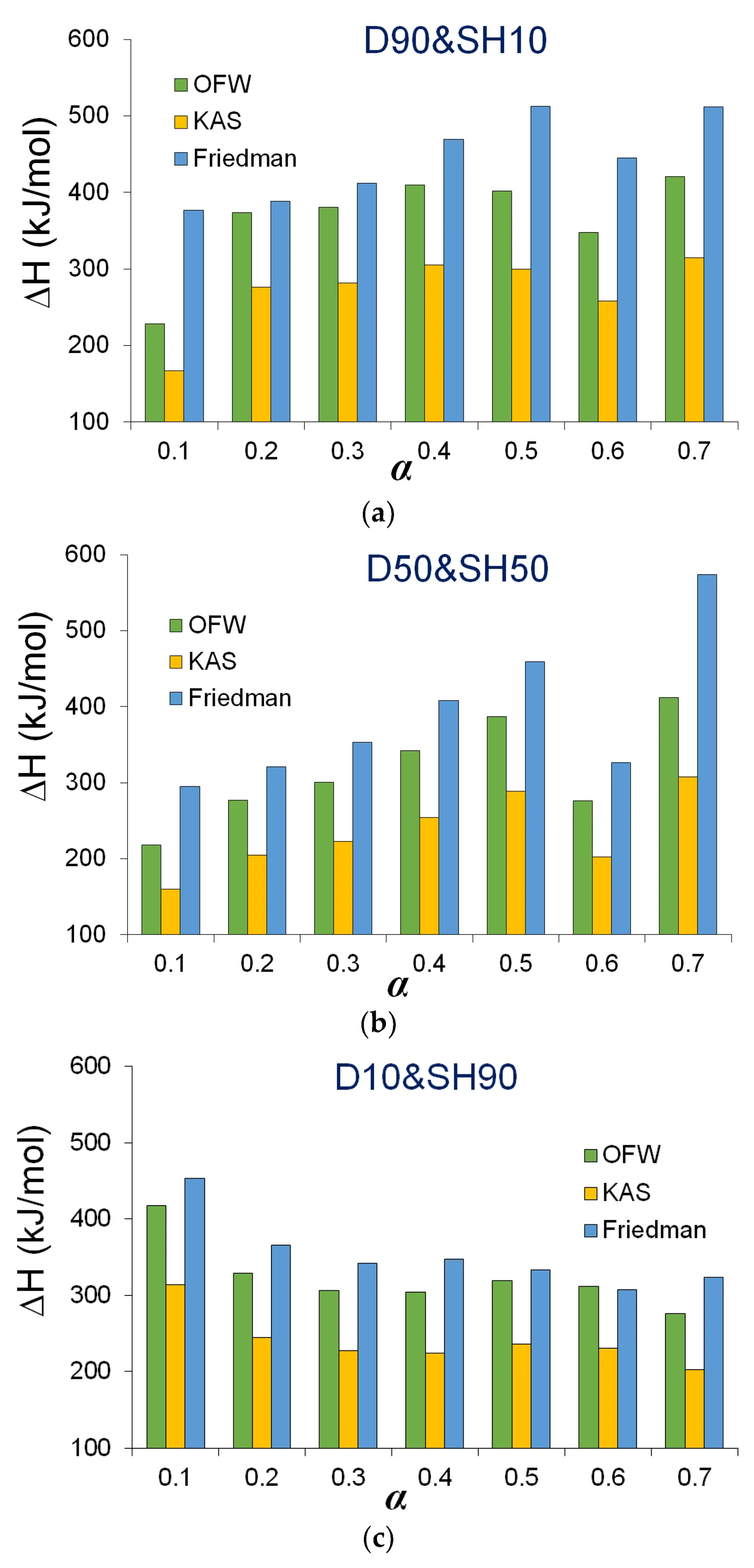
Enthalpy for samples: D90&SH10 (**a**), D50&SH50 (**b**), and D10&SH90 (**c**).

Knowledge of the thermodynamic parameters of a process is essential for determining the viability of thermal and energy processes. The thermodynamic parameters were determined for a heating rate of 10 °C/min. In biomass pyrolysis, enthalpy (ΔH) is the total energy required to decompose the biomass into solid, liquid, and gaseous products. The ΔH values for the studied samples (Figure 5) were in the range of 218.49–420.43 kJ/mol according to the OFW method, 159.98–314.61 kJ/mol according to the KAS method, and 294.70–574.19 kJ/mol according to the Friedman method. Positive ΔH values indicate the endothermic nature of biomass pyrolysis, which implies the need for energy from an external heat source [48]. The difference between the average values of Eα and ΔH is insignificant, less than 5 kJ/mol (for all methods), which indicates favorable conditions for shifting the pyrolysis reactions towards the formation of stable products; therefore, pyrolysis of chicken waste can be carried out in an energy-efficient method [31,53].

**Figure 6:**
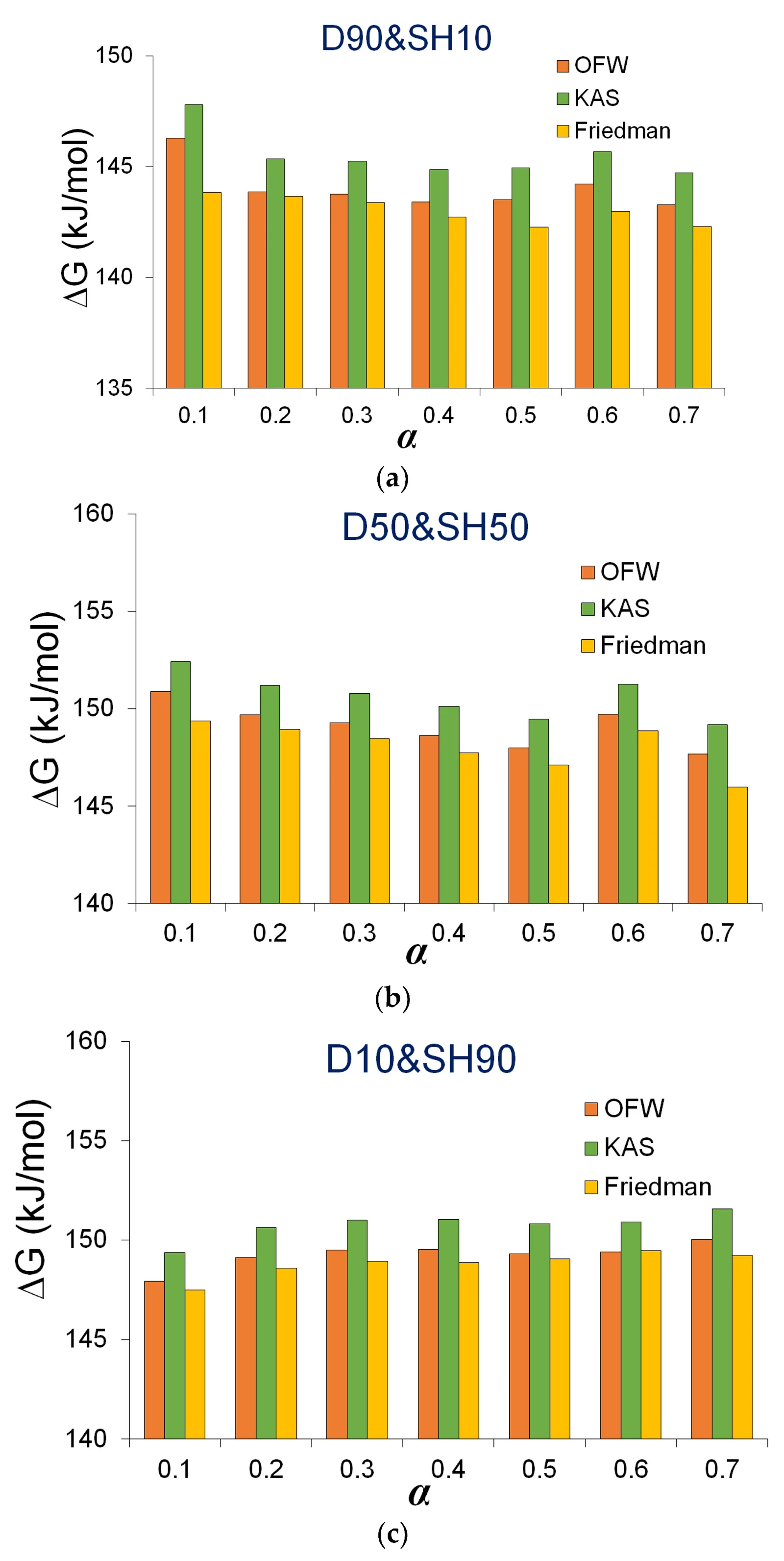
Gibbs free energy for samples: D90&SH10 (**a**), D50&SH50 (**b**), and D10&SH90 (**c**).

Entropy depends on enthalpy and Gibbs free energy (ΔG). The change in ΔG allows us to judge the energy required for thermal decomposition of biomass (Figure 6). The higher the ΔG value, the more difficult it is for the reaction to occur [54]. The average ΔG values for the studied samples ranged from 144.05 to 149.26 kJ/mol according to the OFW method, from 145.52 to 150.76 kJ/mol according to the KAS method, and from 143.02 to 148.81 kJ/mol according to the Friedman method. For most known types of biomass and their mixtures, ΔG values are positive [48,55,56].

**Figure 7:**
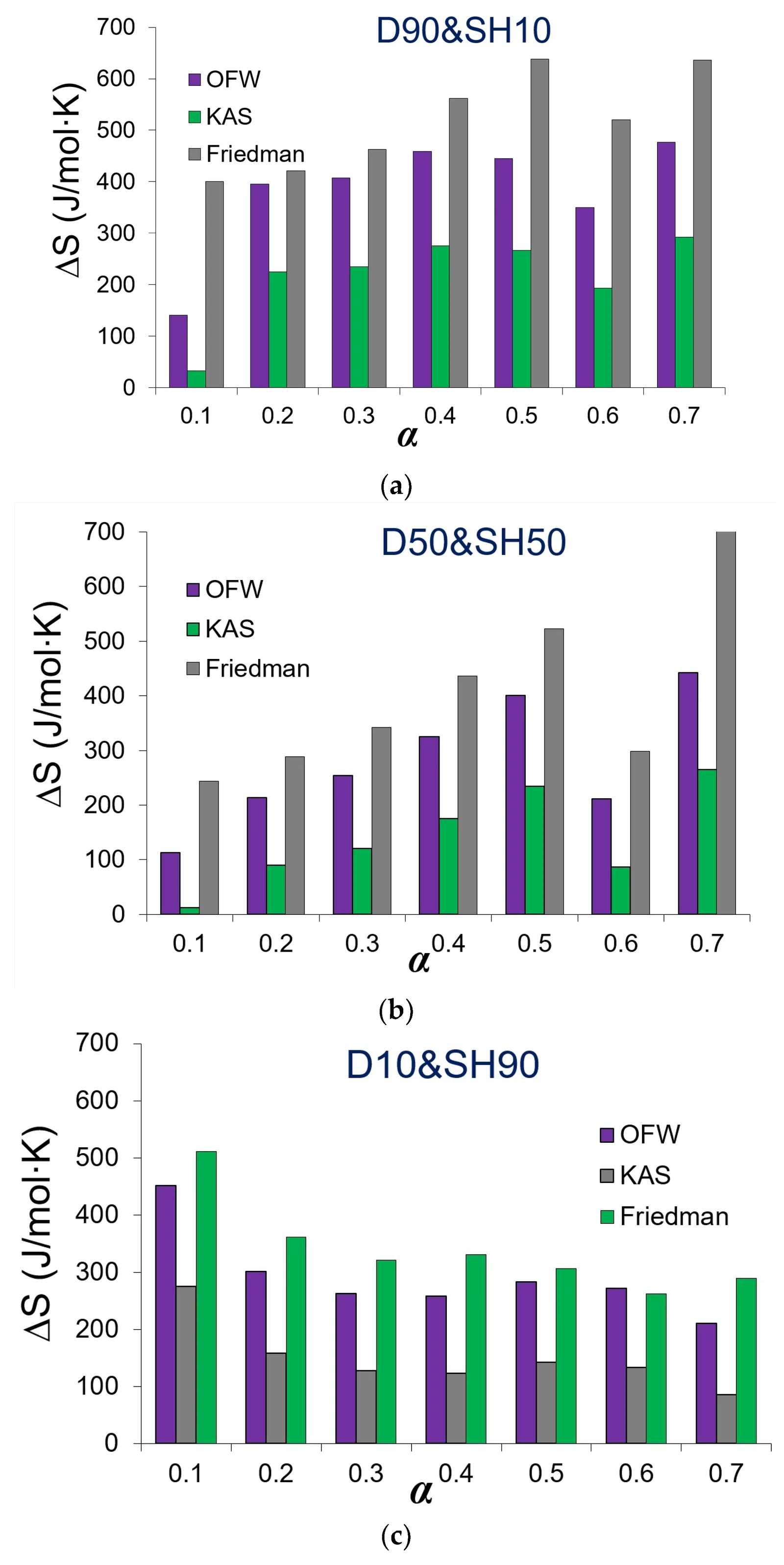
Entropy for samples: D90&SH10 (**a**), D50&SH50 (**b**), and D10&SH90 (**c**).

Entropy (ΔS) is a function of state that represents the degree of disorder of a thermodynamic system. Throughout the pyrolysis process, the ΔS values are positive for all samples, indicating high reactivity of the biomass and rapid formation of the activated complex. The average ΔS values for the studied samples were in the range of 280.07–382.07 J/mol∙K according to the OFW method, 140.37–217.56 J/mol∙K according to the KAS method, and J/mol∙K according to the Friedman method (Figure 7).

### 2.3. Pyrolysis Results in a Laboratory Installation

#### 2.3.1. Material Balance and Pyrolysis Products

The room temperature during the experiment was 25 °C. The evolution of the vapor–gas mixture for D90&SH10 and D50&SH50 began at a temperature of about 180 °C, 15 min after the start of the experiment, which is consistent with the TGA data. For D10&SH90, the onset of the evolution of volatile components was observed at 166 °C, after 14 min. The first drops of pyrolysis liquid were obtained at a temperature of about 310 °C (28 min). The most active evolution of the liquid product for all samples was observed in the temperature range of 330–346 °C. The most intense evolution of the vapor–gas mixture from the pyrolysis reactor is observed for 6 min. Then the process slowed down, and the evolution of the vapor–gas mixture ceased. After 52 min 30 s, a temperature of 550 °C was reached, which is also confirmed by the TGA data. The reactor was maintained at 550 °C for 10 min. The reactor was heated throughout the experiment under a constant nitrogen flow. After this time, the heater was turned off, the reactor was cooled naturally, and the pyrolysis char was removed. The resulting samples were weighed to determine the pyrolysis char yield by mass as a percentage.

**Figure 8:**
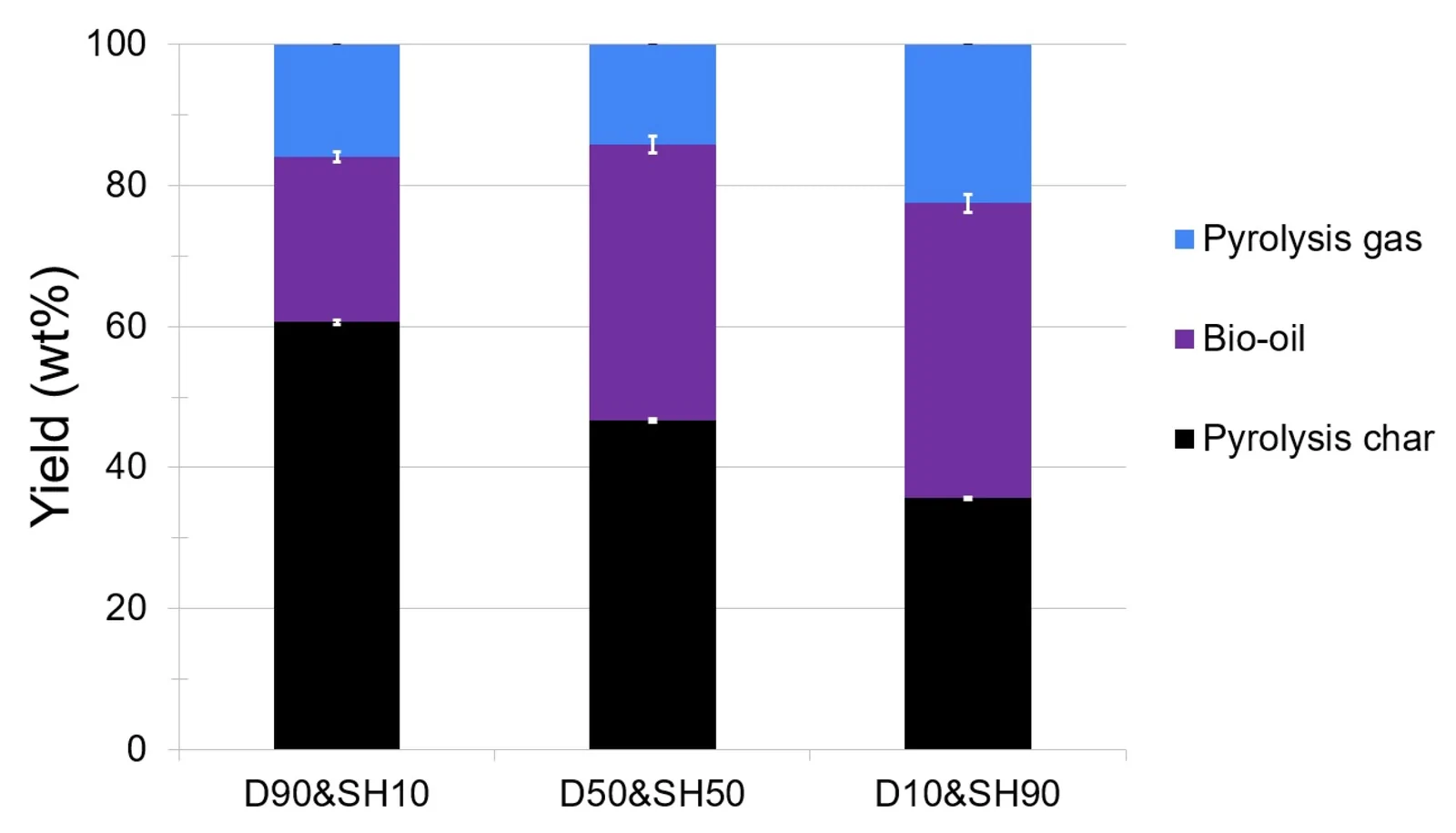
Material balance of the pyrolysis process.

The distribution of the yield of pyrolysis products for three types of samples is shown in Figure 8 and is expressed as a weight percentage.

It is evident that the distribution of the yield of products depends on the content of the initial components of the mixture. The results of the study clearly show that the yield of solid carbonaceous residue decreases with an increase in the proportion of SH in the mixture [31,37]. The addition of SH helps to increase the yield of bio-oil. Pyrolysis of digestate D yields a large amount of solid product, which is attributed to the high ash content. The yield of solid product from D90&SH10 is more than 60 wt%, while the yield of D50&SH50 is about 46 wt% and D10&SH90 is more than 35 wt%. The highest volatile content in SH resulted in the liquid product yield from D10&SH90 of 41.78 wt% compared to other samples, which yielded about 39–42 wt%. Similar results were obtained in [57]. The maximum amount of solid carbonaceous residue obtained from the mixture of chicken manure and rice husk was more than 67 wt%, while the mixture of wood chips provided the highest liquid yield of 44.4 wt% [58,59]. Overall, the obtained results are in good agreement with the TG data and the results of studies by other authors [36,58,59,60].

#### 2.3.2. Physicochemical Properties of the Solid Product

Compared with the feed blends (3.1–13.4 wt%, Table 1), the fixed carbon content of the chars increased to 36.75–46.08 wt% (Table 4), owing to the release of volatiles and the resulting enrichment of the non-volatile carbon, as well as condensation and aromatisation reactions at 550 °C. The main characteristics of the pyrolysis char obtained from the studied biomass are presented in Table 4. The highest content of *FC* was detected in sample D10&SH90.

The main mineral components of pyrolysis char were K, Ca, Fe, Si, and P. Their total content amounted to 95.3%, 98.3%, and 94.2% of the total mass of the mineral part of the samples PC550-2, PC550-1, and PC550-3, respectively (Table 4). It should be noted that the content of potentially toxic heavy metals (As, Cr, Rb) in the samples of pyrolysis char is not significant.

#### 2.3.3. FTIR Spectra of Pyrolysis Char

**Figure 9:**
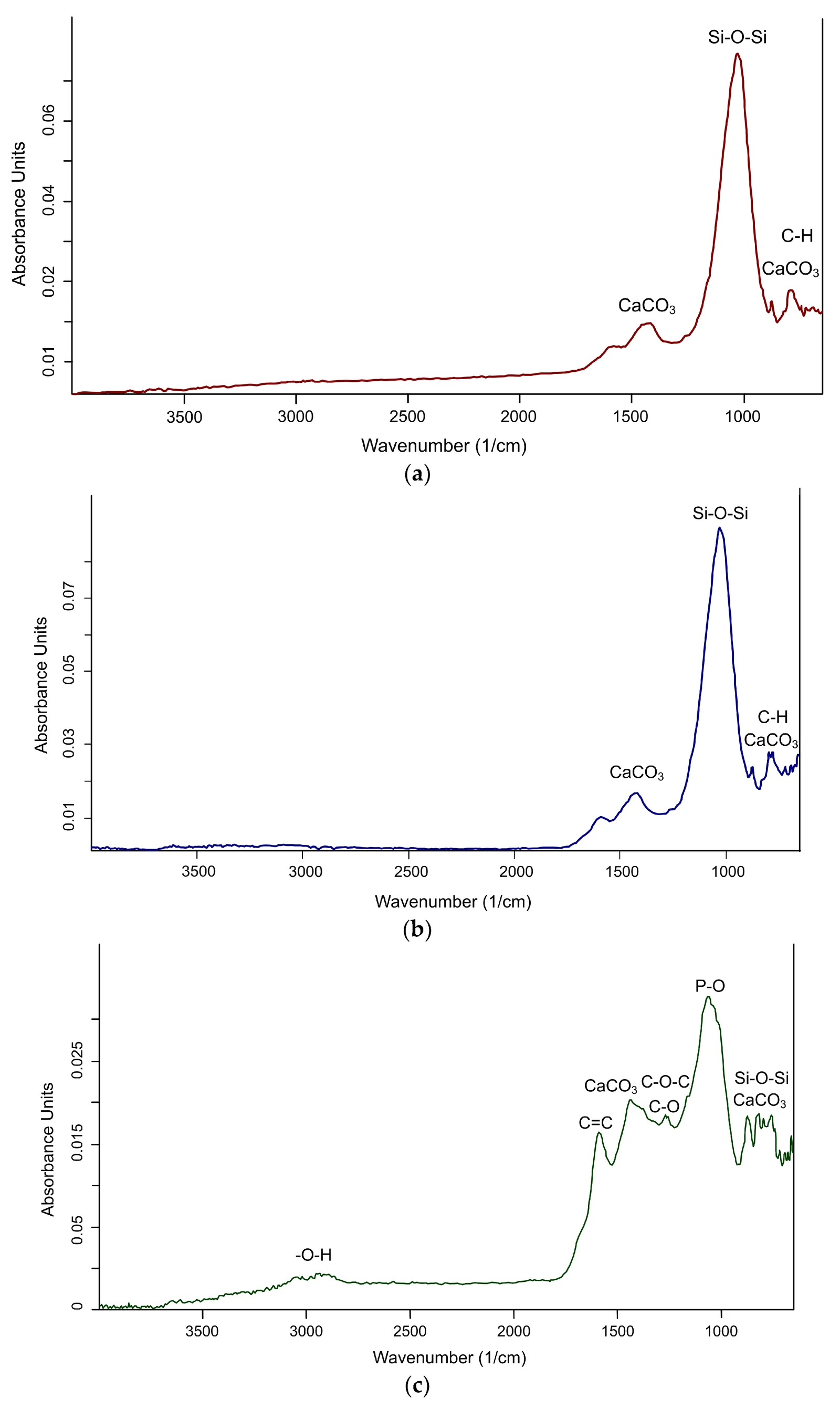
FTIR analysis of biochar samples: PC550-1 (**a**), PC550-2 (**b**), and PC550-3 (**c**).

To determine the functional groups on the surface, an IR spectral analysis was carried out. The results of the sample analysis are presented in Figure 9.

FTIR spectra revealed the presence of various chemical functional groups in the studied samples (Table 5).

Analysis of the FTIR spectra of the obtained samples allows for a detailed characterization of the organic matrix transformation and the evolution of surface functional groups during pyrolysis. Differences in the composition of the initial biomass—the lignocellulosic nature of sunflower husks versus chicken manure, which is rich in proteins, aliphatic compounds, and mineral components—determine the unique spectral profiles of the solid pyrolysis products.

During the co-pyrolysis of sunflower husks and chicken manure, the IR spectra exhibit a superposition of the absorption bands described above. A small band in the 3000–3600 cm^−1^ region of sample PC550 3 corresponds to the stretching vibrations of hydrogen-bonded hydroxyl (–OH) groups in alcohols, phenols, and carboxylic acids. High-intensity peaks in the 1080–1100 cm^−1^ range for samples PC550 1 and PC550 2 correspond to the asymmetric stretching vibrations of Si–O–Si bonds. The IR spectra of sample PC550 3 show a weak signal in this region, attributed to the low silicon mineral content in the sample’s chemical composition [61]. Bands near 1587–1594 cm^−1^ in chars PC550 1 and PC550 3 are associated with C=C stretching vibrations in aromatic rings. The appearance of a peak in the 1400–1440 cm^−1^ region indicates the presence of CaCO_3_ carbonates, which are part of the manure’s mineral (ash) fraction—a component whose concentration increases significantly during carbonization. Peaks in the 1300–1150 and 1050–1150 cm^−1^ regions can be associated not only with C–O–C and C–O bonds but also with asymmetric stretching vibrations of phosphate anions (P=O), confirming the high phosphorus content in samples PC550 1 and PC550 3 [62]. Peaks in the 754–772 cm^−1^ range indicate out-of-plane bending vibrations of C–H bonds in benzene (aromatic) rings—characteristic of lignin—as well as vibrations of the pyranose ring in carbohydrates (cellulose, hemicellulose). The FTIR analysis of the samples is consistent with other data on solid pyrolysis products derived from various types of biomass [63,64].

#### 2.3.4. Composition of the Liquid Product

As a result of the analysis of the organic component of the pyrolysis liquid using GC-MS for PL550-1, 104 organic compounds were identified, which accounts for 57.12% of the total composition of the fraction (see Table 6).

For sample PL550-2, mass spectra were obtained for 83 substances of organic origin (75.05%). In turn, analysis of the pyrolysis liquid of the third composition, PL550-3, revealed 86 organic substances (75.43%). The detected organic substances were grouped into three categories: hydrocarbons, oxygen-containing substances, and nitrogen-containing substances. Other (unidentified) compounds account for 43.26%, 27.95%, and 24.57% for the studied formulations PL550-1, PL550-2, and PL550-3, respectively.

The detected hydrocarbons were products of the primary and secondary cracking of the feedstock’s aliphatic chains. The presence of unsaturated (alkenes) and aromatic hydrocarbons indicates that dehydrogenation, cyclization, and aromatization processes occurred at a temperature of 550 °C. It is known that a high hydrocarbon content increases the calorific value of liquid fuel [53]. Oxygenated compounds in all samples consisted of phenols, carboxylic acids, ketones, aldehydes, alcohols, and esters. The formation of phenols is associated with the thermal degradation of lignin or aromatic polymers, while carboxylic acids are products of the degradation of hemicellulose or lipid components. Hexanoic acid, propanoic acid, butanoic acid, and pentanoic acid were identified in the pyrolysis liquid samples. Ketones, aldehydes, alcohols, and esters are formed as a result of the breakdown of carbohydrate macromolecules [36]. Nitrogen-containing compounds in the pyrolysis liquids of samples PL550-1 and PL550-2 consisted of amides, nitriles, and heterocycles. These are formed through the thermal decomposition of nitrogen-containing components in the feedstock; in the case of chicken manure, these may include protein and amino acid components [65]. Sample PL550-1 is characterized by the highest total hydrocarbon content (20.66%), dominated by saturated aliphatic compounds (10.43%) and aromatic compounds (5.62%). In samples PL550-2 (4.97%) and PL550-3 (7.98%), the proportion of hydrocarbons decreases sharply. This indicates that in the first case, conditions favored more destructive cracking with the formation of stable hydrocarbon radicals, whereas in the second and third compositions, degradation shifted toward oxidative processes. Oxygen-containing compounds constitute the bulk of the identified liquid phase in all samples, yet their distribution varies significantly:

A pronounced linear increase in carboxylic acid content is observed, rising from PL550-1 (4.56%) to PL550-2 (17.98%) and reaching PL550-3 (26.73%). Such a high acid concentration in PL550-3 points to the intensive cleavage of oxygen-containing bonds in the feedstock, rendering this sample the least suitable for direct use as fuel without prior upgrading.

Phenol content remains consistently high across all three samples, showing a moderate increase (from 14.89% in PL550-1 to 20.39% in PL550-3). This confirms the similar nature of the aromatic framework within the initial matrix across all compositions, consistently yielding phenolic monomers at 550 °C. Sample PL550-2 exhibits elevated concentrations of ketones (11.08%) and alcohols (5.63%), indicating a specific thermal decomposition pathway for carbohydrate fractions that slows down at the stage of carbonyl compound formation. A notable distinguishing feature is the complete absence of nitrogen-containing compounds in sample PL550-3. Conversely, their proportion is highest in PL550-1 (7.53%) and drops to 3.37% in PL550-2. This trend indicates that the feedstock composition or the conditions under which PL550-3 was obtained facilitated the complete transfer of nitrogen into the gas phase.

Thus, sample PL550-1 represents the most “fuel-oriented” fraction, characterized by a high hydrocarbon content and low acid content. In contrast, sample PL550-3 is a highly oxidized chemical mixture dominated by organic acids and phenols; while it holds significant potential for the extraction of valuable chemical substances (feedstock for organic synthesis), it requires deoxygenation before it can be processed into fuel compositions.

## 3. Discussion

Previously, the research group analyzed the processes of anaerobic digestion and pyrolysis separately and jointly [27,50]. A preliminary study was conducted on biogas production from poultry waste in an anaerobic digestion unit (chicken manure). The digestate obtained during laboratory studies requires further processing into valuable products due to its toxicity and the need for comprehensive disposal. Biogas production processes are characterized by incomplete conversion of the feedstock, making digestate a promising material for energy production [13]. Preliminary studies were conducted to obtain information on the characteristics of the thermal decomposition of samples under pyrolysis conditions (argon, 39–1000 °C) [30]. Pyrolysis of poultry manure demonstrated a multi-stage destruction process. Thermal decomposition at the active pyrolysis stage is characterized by three peaks corresponding to temperatures of 281 °C, 333 °C, and 427 °C. The experiments revealed that the initial characteristics of chicken manure meet the requirements for raw materials for processing in thermochemical conversion units.

The decomposition of SH in an inert environment is characterized by the stages of conversion of three main components (hemicellulose, cellulose, lignin), which occur in parallel, but at different rates over a wide temperature range. Chicken manure, in contrast to SH, is characterized by a wider temperature range of the volatile matter release process, which is associated with the rapid oxidative decomposition of organic compounds in the material, such as carbohydrates and proteins, with the formation of volatile compounds and the conversion of lignocellulosic components of organic matter [28,38,52].

The results of this study were necessary for understanding the kinetics of chemical reactions occurring during the thermochemical conversion of complex heterogeneous feedstocks in order to determine safe pyrolysis conditions [34,36]. TGA and kinetic analysis data indicate the complexity of the D and SH pyrolysis process, accompanied by multiple sequential and parallel reactions. High variability in *E_α_* values was observed for the mixture [28,29]. These results, combined with observations from other authors, indicate that the samples under study consist of multicomponent biomass; consequently, their thermal decomposition is governed by more than one energy barrier. Thermogravimetric analysis revealed that, despite the materials’ diverse origins and heterogeneous nature, common features can be identified in the decomposition of hemicellulose and cellulose.

An analysis of the thermal decomposition data obtained in this study, alongside results from other research, showed that the initial mass loss due to the removal of adsorbed moisture is typical for all types of biomass and mixtures containing livestock and poultry waste [34,36]. The second stage was characterized by the active pyrolysis of proteins, cellulose, and hemicellulose. Analysis of the TG and DTG curves revealed that mixing biomass with chicken manure (sample D) smooths the DTG curve, as the decomposition peaks for hemicellulose and manure proteins overlap with the cellulose decomposition peak of the biomass [30]. Regarding the third stage of thermal decomposition (above 550 °C), the observed peaks or the slowing of mass loss are attributed not only to lignin degradation but also, presumably, to the decomposition of inorganic carbonates (calcium and potassium)—compounds initially present in high concentrations in the chicken manure [46,48].

Kinetic analysis using the OFW, KAS, and Friedman methods revealed that the co-pyrolysis mechanism of the samples is quite complex due to their heterogeneous composition. The activation energy for all samples is quite high. The increase in energy activation values at the end of the pyrolysis process can be explained by the low volatility and high structural stability of lignin [49], as well as changes in the pyrolysis mechanism and catalysis of metals contained in the ash [27,29].

Activation energy values obtained using isoconversional methods, as well as thermodynamic parameters for the pyrolysis of biomass, manure, and chicken litter, span a wide range depending on the feedstock composition and the degree of conversion. Comparative data are presented in the table and align with other studies.

In [43], the authors suggest that elevated activation energy values at high conversion levels may be attributed to either the mineral/ash catalytic effect or the formation of condensed lignin-like aromatic char structures; alternatively, both mechanisms may contribute to the activation energy values. Consequently, further research is required to fully understand and describe the kinetics and mechanism of thermal decomposition. Overall, the good agreement among all three models, combined with robust values *R^2^*, confirms the validity of the isoconversional approach for describing the pyrolysis kinetics of the studied mixtures containing chicken litter. The thermodynamic parameters of thermal decomposition for the investigated mixtures also align well with values reported for various types of biomass (see Table 7).

During the research, solid and liquid pyrolysis products were characterized using a laboratory setup, providing a scientific basis for their potential practical applications. Analysis of the pyrolysis char properties revealed that an elevated potassium (K) concentration is characteristic of both manure and lignocellulosic biomass [9,10,13,50]. The highest content of major mineral components (K, Ca, Fe, Si, and P) was found in sample PC550-3, while the lowest was in sample PC550-1. The composition of chicken manure is not uniform and depends significantly on the poultry’s diet, the type of bedding, and other factors. During the anaerobic digestion of manure, a portion of the mineral components transfers to the liquid phase and is removed during separation, which can also reduce their concentration in the resulting char.

IR spectroscopy of the pyrolysis char samples (Table 5) revealed the presence of specific O–H, C=C, C–O, P=O, and Si–O–Si groups, indicating the potential for using these materials as sorbents, fertilizers, and fillers [14]. The high content of oxygen-containing groups and aromatic rings in samples PC550-1 and PC550-3 accounts for their sorption properties. The presence of both aromatic domains (derived from straw) and residual nitrogen–phosphorus groups and carbonates (derived from manure) in the mixed biochar makes it a promising soil amendment capable of ensuring prolonged nutrient retention [79,80]. The Si–O–Si groups present in sample PC550-2 are suitable for the creation of new composite materials. This opens up possibilities for producing bio-fertilizers that contain more stable mineral forms and possess a robust structure for retaining them [81,82].

According to the data obtained for assessing the composition of the liquid pyrolysis product, a direct correlation was found for oxygenated compounds as the SH content increased from 10 to 90 wt%. Thus, a characteristic increase was observed from 28.55% for PL550-1 to 63.71% and 67.45% for PL550-2 and PL550-3, respectively. Particular attention should be paid to the group of carboxylic acids. With a decrease in the proportion of D in the mixture from 90% to 50%, the content of carboxylic acids increases fourfold. An increase is observed from 4.56% (PL550-1) to 17.98% in PL550-2 and 26.73% in PL550-3. A slight increase in the content of carboxylic acid esters and aldehydes is also noted.

The phenol content increases from 14.89% to 20.39% as the proportion of SH rises, since phenols are characteristic products of the decomposition of husk lignin [54,70]. At the same time, the relatively high phenol content observed even in PL550-1 (10% SH) points to an additional source: the decomposition of aromatic amino acids (tyrosine) from manure proteins, a process that yields phenol and cresols. The most pronounced changes are noted for nitrogen-containing substances, including amides, nitriles, and heterocyclic compounds. When the D content decreases from 90% to 50%, its content drops from 7.53% to 3.37%, and in the PL550-3 sample. The high digestate content leads to the formation of nitrogen-containing pyrolysis products, which is explained by the fact that chicken manure contains more nitrogen than sunflower husks [1,31].

The resulting pyrolysis liquids, after purification and fractionation, can be used as a component of boiler fuel or as a feedstock for the chemical industry. For use as a fuel, it is necessary to isolate the hydrocarbon component and remove the water component and oxygen-containing compounds. Phenols, alcohols, and aromatic hydrocarbons can serve as a source of feedstock for the production of resins, plastics, and solvents. SH-dominated co-pyrolysis (samples D10&SH90) is characterized by a small region with a positive synergistic effect. In the low-temperature range for this sample, chemical decomposition and depolymerization reactions played a dominant role in the co-pyrolysis process.

This study investigated the potential for a positive or negative synergistic effect arising from the interaction between the components D and SH. In this study, conclusions regarding synergy are limited to the conditions of 10 °C/min. Analysis of the TG curves reveals a positive synergistic effect within narrow temperature ranges associated with the biomass’s contribution to the mixture’s thermal degradation; this effect is particularly pronounced for the D10&SH90 sample. At other stages, the mixtures under study can be regarded as essentially inert carbonaceous substrates, indicating that the free-radical-donating effect of the biomass plays no significant role. The samples possess distinct chemical structures, and upon mixing, the individual components may inhibit the decomposition process. A positive synergistic effect during co-pyrolysis was also determined in a mixture of chicken manure and plastic [47,83]. Similar inhibitory effects observed during the conversion of various types of biomass are reported in other studies [10,29]. At the same time, the heating rate plays a crucial role as a parameter governing product distribution during co-pyrolysis by influencing the temperature [83]. Study [84] suggests that adding plant biomass to chicken manure increases the yield of tar and char at low heating rates. Consequently, selecting an optimal heating rate to obtain the desired products also allows one to capitalize on the benefits of adding a plant component to complex poultry waste.

This positive effect is confirmed by experimental data obtained using a laboratory setup (Figure 8) and is manifested as an increase in the volume of evolved volatiles and pyrolysis gas. Therefore, further research into the co-pyrolysis of the studied samples at various heating rates is required to determine the optimal reaction conditions within the pyrolysis reactor.

Undoubtedly, obtaining mixtures through various variations in the utilization of chicken manure digestate allows for the removal of pathogenic microorganisms and other harmful substances that increase the risk of environmental pollution during thermochemical treatment [15]. At the same time, the total volume of waste from the biogas plant is significantly and quickly reduced, yielding convenient bioenergetic resources that can be used primarily directly for the energy needs of the pyrolysis plant itself, or for drying wet digestate in the case of a combination of technologies.

## 4. Materials and Methods

In this study, experimental pyrolysis studies were conducted on a laboratory setup, and thermogravimetry was also used to investigate thermochemical conversion. OFW, KAS, and Friedman methods based on TGA data were used to evaluate kinetic parameters. Proximate and ultimate analyses of the studied samples will allow us to evaluate their potential for use as high-value-added products.

### 4.1. Sample Materials

**Figure 10:**
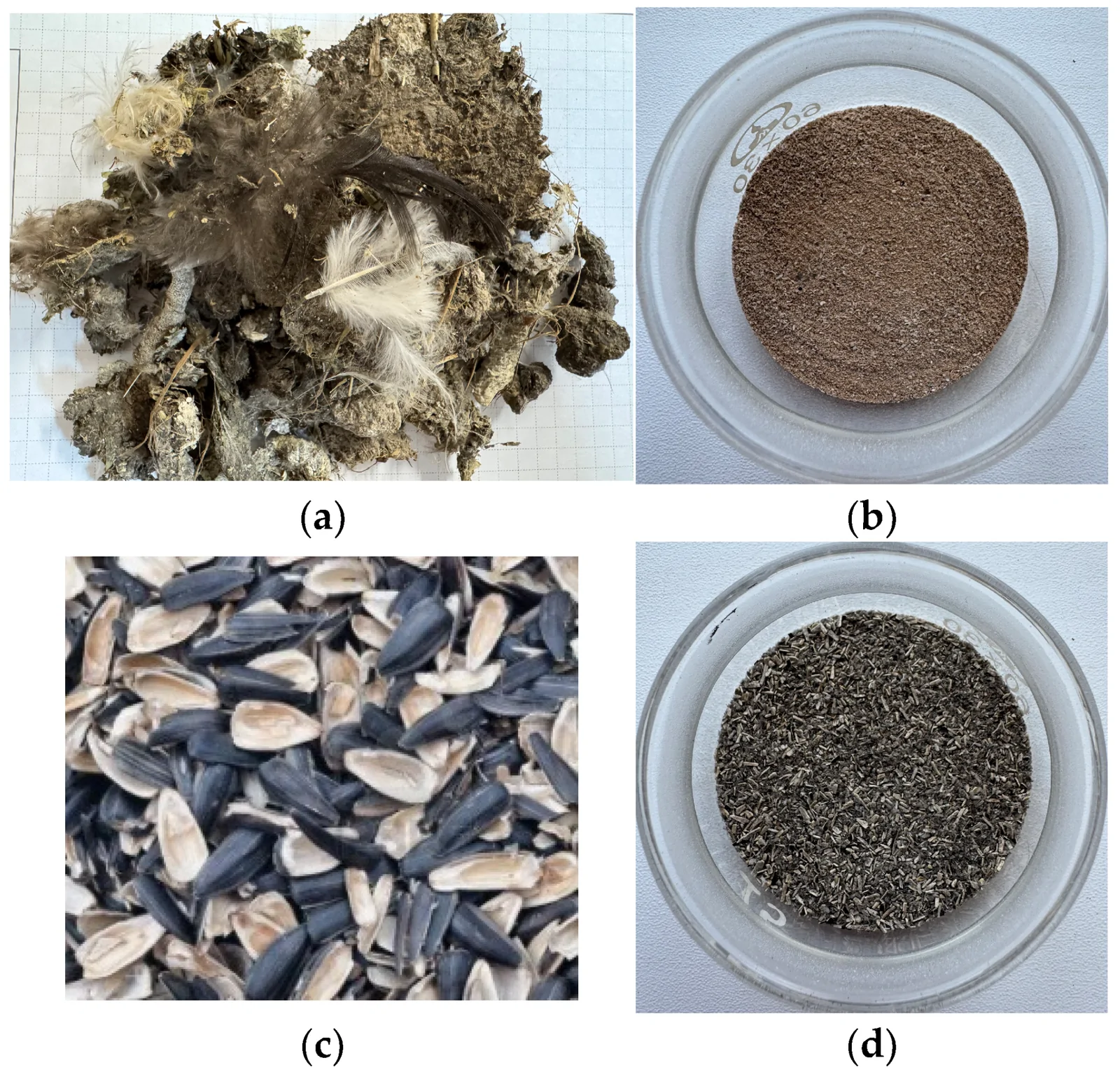
Photographs: untreated chicken manure of chicken manure (**a**), digestate (**b**), untreated SH (**c**), crushed and dried SH (**d**).

The samples were prepared from a mixture of digestate obtained from anaerobic digestion of chicken manure D with the addition of sunflower husks SH (Kazan, Russia). Photographs of the feedstock samples (before anaerobic digestion and processing) are shown in Figure 10.

After completion of the anaerobic digestion stage, D was collected with a moisture content of 90%. Drying of D involved dehydration using a drying apparatus at 105 °C to remove moisture for 24 h. To conduct studies on thermochemical conversion, the biomass was crushed to a fraction of less than 0.2–0.5 mm (from 200 to 500 µm). SH had a crude fat content of 3–5% (absolute dry content) and a crude fiber content of 55–70% (absolute dry content). Before the experiment, SH was dried at 105 °C for 1 h and stored in sealed plastic bags for further experiments. Before mixing, each sample was carefully weighed using an analytical microbalance. Binary mixtures for the study were prepared by varying the mass fractions of D and SH. All crushed samples were stored in airtight containers to prevent moisture absorption and for use in further experiments.

### 4.2. Physicochemical Characterization

Elemental analysis was performed on a Euro EA3000 elemental analyzer (Eurovector, SpA, Milan, Italy). Oxygen content (O, wt% based on air-dry weight) and higher heating value (*HHV*) of the samples were calculated using standard formulas (1, 2 and 3) [55,71,85]. Determination of sulfur in this device was impossible due to technical features. Standard methods were used for technical analysis [86,87,88,89].

The oxygen (O) content was computed using equation:

O = 100 − (C + H + N +*Ash*), (1)

The fixed carbon (*FC*) content was calculated using the following equation:

*FC* = 100 − (*Moisture* + *VM* + *Ash*), (2)

The *HHV* (MJ/kg) of the samples were determined using equation:

*HHV* = 0.3491 ⋅ C + 1.1783 ⋅ H − 0.1034 ⋅ O − 0.0151 ⋅ N − 0.0211 ⋅ *Ash* (3)

### 4.3. Thermal Analysis

#### 4.3.1. TGA

TGA was performed using a synchronous microthermal analyzer STA 449 F3 Jupiter (Netzsch, Selb, Germany). All studies were carried out in an argon atmosphere with a flow rate of 50 mL/min. The sample was placed in a crucible and heated from room temperature to 700 °C. Four heating rates were investigated during the experiments: 5, 10, 20, and 30 °C/min. The representativeness of a fuel mixture weighing several milligrams depends on its homogeneity. For the studies, the samples was ground and sieved to a particle size of less than 500 microns. A preliminary assessment of the microstructure of the mixtures was carried out using scanning electron microscopy. The reliability of the analysis was achieved by conducting a series of parallel measurements (three replicates), taking into account statistical assessment of the data dispersion. The error in repeating the experiment was 1.5%.

#### 4.3.2. TG-FTIR

To measure the IR spectra of gaseous products, a Fourier-transform infrared spectrometer (Bruker Optik GmbH, Ettlingen, Germany) was used.

#### 4.3.3. Study of Synergistic Effects in Co-Pyrolysis

For the co-pyrolysis of mixtures, it is important to demonstrate a synergistic/non-additive effect, since the mixtures behave differently than the weighted sum of their components during co-processing [56]. Synergism can be measured by comparing the theoretical TG/DTG curve (*TG_calc_*) with the experimental TG/DTG curve (*TG_exp_*) for the pyrolysis of the mixtures under consideration. The theoretical curve can be obtained by summing up the pyrolysis results of the initial components of the mixtures according to the composition of the feedstock [59].

#### 4.3.4. Kinetic Analysis

Thermal conversion of biomass produces a solid carbonaceous residue and volatile substances. This process is described by a general transformation scheme, which can be expressed by the following equation:

(4) $$\mathrm{Biomass}\overset{k(T)}{\to }{\mathrm{Solid}\;\mathrm{residue}\;+\;\mathrm{Volatiles}}_{\mathrm{condencable}\;+\;\mathrm{noncondencable}}$$

For non-isothermal experiments of thermal decomposition of a solid at a linear heating rate *β* = *dT*/*dt*, the generalized fundamental equation has the form:

(5) $$\frac{\mathrm{d\alpha}}{\mathrm{dT}}=\frac{A}{\beta }\cdot \exp\left(-\frac{E_{\alpha }}{R\cdot T}\right)\cdot f\left(\alpha \right)$$

where *E_α_* is the activation energy (kJ/mol); *T* is temperature (K); *R* is the universal gas constant (8.314 J/mol∙K); *A* is the pre-exponential factor (1/s).

Integration of Equation (5) allows for the analysis of kinetic data. Integration can be performed using isoconversion (model-free) methods, which estimate the activation energy as the degree of conversion changes and do not require any prior knowledge of the decomposition reaction mechanism.

The OFW method allows one to calculate activation energy values without a known reaction model based on the rate of thermochemical conversion at several heating rates. The equation for calculating activation energy is as follows [57]:

(6) $$\ln\left(\beta_{i}\right)=\ln\left(\frac{A\cdot E_{\alpha }}{R\cdot g\left(\alpha \right)}\right)-5.331-1.052\frac{E_{\alpha }}{R\cdot T}$$

The KAS method for calculating activation energy is as follows:

(7) $$\ln\left(\frac{\beta_{i}}{T_{\mathrm{\alpha i}}^{2}}\right)=\ln\left(\frac{A_{\alpha }R}{E_{\alpha }\;g\left(\alpha \right)}\right)-\frac{E_{\alpha }}{RT_{\mathrm{\alpha i}}},$$

where *g*(*α*) is constant with given conversion value.

The Friedman method is expressed as follows:

(8) $$\ln\left[\beta_{i}{\left(\frac{\mathrm{d\alpha}}{\mathrm{dT}}\right)}_{\alpha ,i}\right]=\ln\left[A_{\alpha }f\left(\alpha \right)\right]-\frac{E_{\alpha }}{\mathrm{RT}},$$

where the subscript *i* is given heating rate value, and subscript *α* is given conversion degree.

#### 4.3.5. Thermodynamic Analysis

Estimation of thermodynamic parameters is a useful tool for understanding the co-pyrolysis process. The changes in the parameters ∆H (kJ/mol), ∆G (kJ/mol), and ∆S (J/mol∙K) were calculated using the following formulas [58]:

Δ*H* = *E_α_* − *R* ⋅ *T_peak_*, (9)

(10) $$\Delta G=E_{\alpha }+\left(R\cdot T_{\mathrm{peak}}\cdot \ln\frac{K_{B}\cdot T_{\mathrm{peak}}}{h\cdot A}\right),$$

(11) $$\Delta S=\frac{\Delta H-\Delta G}{T_{\mathrm{peak}}},$$

where *T_peak_* is the temperature corresponding to the maximum mass loss rate, °C; *K_B_* is Boltzmann constant (1.38∙10^−23^ J/K); *h* is Planck’s constant (6.626∙10^−34^ J∙s).

### 4.4. Experimental Pyrolysis Procedure Using a Laboratory Setup

**Figure 11:**
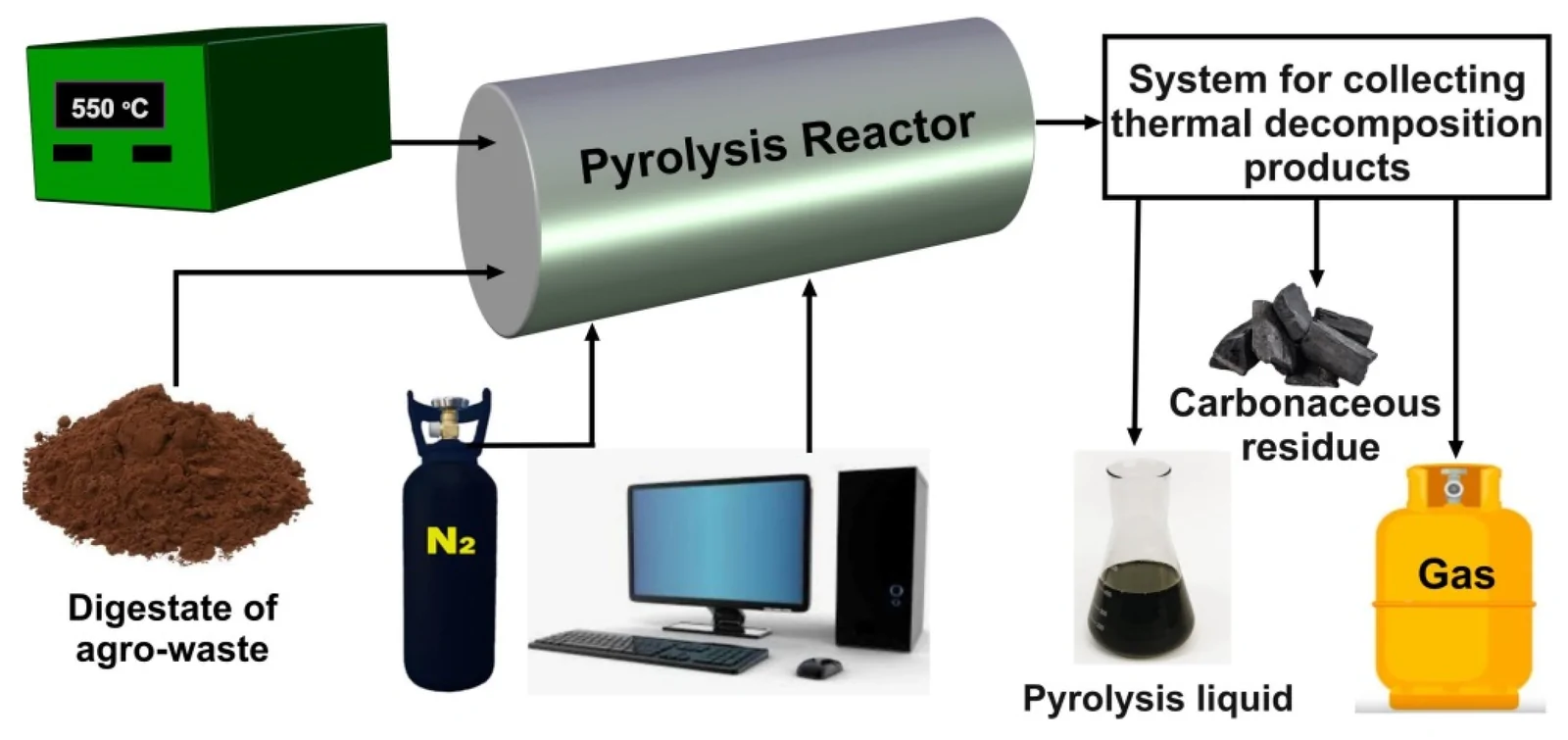
Scheme of the pilot scale setup for studying the pyrolysis process.

Pyrolysis was carried out on a laboratory setup (Institute of Power Engineering and Advanced Technologies, Russia) (Figure 11) [50].

The setup was a metal tubular reactor with a length of 280 mm and an internal diameter of 34 mm. A 60 g sample was placed in the metal tubular reactor. Samples were weighed with an accuracy of 0.0001 g. The initial pyrolysis temperature was 25 °C, the heating rate was 10 °C/min, and the temperature of the pyrolysis process itself was 550 °C. All measurements were carried out in an atmosphere of inert nitrogen at a constant gas flow rate of 20 L/h.

During pyrolysis, the reactor was placed in an oven. The outlet pipe was connected to a condensation system to remove gaseous and liquid products. Temperature changes were monitored and recorded using a thermocouple connected to software. The thermocouple voltage was controlled and recorded by the data logging program, which allowed for accurate tracking of temperature changes over time.

The samples obtained from the mixture of digestate and sunflower husk additives were subjected to controlled thermal treatment using a single-stage heating protocol. The holding time of the samples in the tubular reactor was about 2 h to ensure complete carbonization. After cooling to room temperature in a nitrogen flow, the resulting pyrolysis char was collected and labeled in accordance with the applied pyrolysis temperature:

PC550-1—pyrolysis char D90&SH10.

PC550-2—pyrolysis char from sample D50&SH50.

PC550-3—pyrolysis from sample D10&SH90.

All pyrolysis chars were carefully weighed and stored in airtight containers. The non-condensable gas was collected in a gas bag, and the pyrolysis liquid was placed in a flask. The liquid oil yield was further characterized to study its composition. The pyrolysis liquid was labeled in accordance with the pyrolysis temperature used:

PL550-1—pyrolysis liquid from sample D90&SH10.

PL550-2—pyrolysis liquid from sample D50&SH50.

PL550-3—pyrolysis liquid from sample D10&SH90.

All experiments were repeated three times, and the averaged mass balance data are presented in the article.

The proportion of liquid oil and solid substance was estimated based on their mass. The material balance was determined using the method presented in [50].

(12) $$W_{\mathrm{carb}.r}=\frac{W_{2}-W_{1}}{W_{\mathrm{feed}}}\cdot 100$$

(13) $$W_{\mathrm{liquid}}=\frac{W_{4}-W_{3}}{W_{\mathrm{feed}}}\cdot 100$$

*W_gas_* = 100 − (*W_liquid_* + *W_carb.r_*) (14)

where *W_feed_* is the weight of sample, g; *W*_1_ is the mass of empty reactor before pyrolysis, g; *W*_2_ is the mass of reactor after pyrolysis, g; *W*_3_ is the mass of empty flask, g; *W*_4_ is the mass of flask with liquid product, g; *W_carb.r_* is the yield of pyrolysis char, wt%; *W_liquid_* is the yield of pyrolysis liquid, wt%; *W_gas_* is the gas yield, wt%.

#### 4.4.1. Physicochemical Properties of Pyrolysis Products

The physicochemical properties of two important co-pyrolysis products have been studied and described: pyrolysis liquid (resin/oil) and pyrolysis char, obtained during the thermal decomposition of the studied mixtures. In this study, the composition of the pyrolysis gas obtained in the laboratory setup was not determined, but, as is known from the literature, the composition of the pyrolysis gas includes CO, CO_2_, CH_4_, and other hydrocarbons. The gases released during TGA were examined using infrared spectroscopy.

#### 4.4.2. Physicochemical Properties of Pyrolysis Char

Ash, and volatile matter content were determined in a muffle furnace (PMLS-2/1200, Milaform, Kazan, Russia) in accordance with standards [86,87,88,89]. X-ray fluorescence analysis experiments were performed on a Clever C3 energy dispersive X-ray fluorescence spectrometer (LLC Eleran, Moscow, Russia) with the following main characteristics: the range of detected Na-U elements, the anode material Rh, the thickness of the beryllium window 0.125 mm, tube voltage 5–50 kV, tube current 20–1000 mA, maximum power 50 W, high-resolution detector-electrically cooled devices on a Peltier element, detector resolution of 135 eV. The elemental composition was determined under the following conditions: medium—vacuum tube voltage—45 kV tube current—200 mA spectrum acquisition time—200 s area of the diaphragm setting the size of the X-ray beam—16 mm^2^. The sample under study was placed in a polypropylene measuring cell with a 0.6-μm-thick polyethylene terephthalate film stretched across the bottom; the film was transparent to X-ray radiation. The presence of elements in the sample under study was determined based on the signal from the characteristic lines (Kα) of each element.

#### 4.4.3. IR Spectroscopic Analysis

IR spectra in the range (400–4000 cm^−1^) were obtained on a Bruker Vertex 70 FTIR spectrometer, with the cuvette compartment purged with dry, purified air to remove water vapor. Interferograms were recorded with a resolution of 2 cm^−1^ and averaged over 128 scans. Spectra of solid samples were recorded using an ATR attachment with a ZnSe crystal (MIRacle, PIKE Technologies, Madison, WI, USA).

#### 4.4.4. Composition of Pyrolysis Liquid

The composition of the organic portion of the pyrolysis liquid was analyzed by chromatography-mass spectrometry using an HP-5MS column (0.25 μm, 30 m, Shimadzu, Kyoto, Japan) and a spectrometer (GCMS-QP2010, Shimadzu, Kyoto, Japan). The organic portion of the pyrolysis liquid was diluted with methylene chloride. The mass spectra of the products were compared with the NIST 2020 library, and the compounds with the highest similarity were considered. In this study, the pyrolysis liquid composition was presented as a percentage of GC-MS peak area, with compounds with an area greater than 1% reported. Peak area values indicate the amount of a given compound, and relative peak areas indicate the relative abundance in the product composition.

## 5. Conclusions

In this study, the co-pyrolysis of digestate and plant residues was investigated using a laboratory slow pyrolysis reactor and TGA. Three degradation stages were observed in the D90&SH10, D50&SH50, and D10&SH90 samples, which were associated with the degradation of lignocellulose components and proteins in chicken manure waste. The highest activation energies were found in the D90&SH10 sample: 367.32 kJ/mol (232.77–414.88 kJ/mol), 274.95 kJ/mol (173.14–311.70 kJ/mol), and 445.87 kJ/mol (381.29–517.76 kJ/mol) according to the OFW, KAS, and Friedman methods, respectively. The average activation energy values for the D90&SH10 and D50&SH50 samples indicate high energy requirements for the co-pyrolysis process. Consequently, this complicates the reactor design, leading to increased capital costs for its fabrication.

The yield of co-pyrolysis products depends on the ratio of the initial mixture components. Pyrolysis gas is low-calorific, as it consists mainly of the non-flammable component CO_2_ according to the TG-FTIR spectra (for all samples). The sample with a high digestate content provides the highest yield of pyrolysis char due to its high ash content. For the D90&SH10 mixture, the yield of pyrolysis char exceeds 60 wt% (PC550-1), and for D10&SH90, it exceeds 35 wt% (PC550-3). The content of FC and mineral elements (K, Ca, Fe, Si, and P) in the solid pyrolysis products increases, making this material a promising additive for soil application. The high VM content of the D10&SH90 sample ensures the highest yield of pyrolysis liquid PL550-3 (41.78 wt%). However, the liquid product PL550-1 is distinguished by the highest hydrocarbon content (20.66%), making it promising for use as a feedstock for upgrading or for the recovery of chemical products.

## Figures and Tables

**Table 1:** Proximate and ultimate analysis of samples.

Samples	Ultimate Analysis (wt%, on Air Dry Basis)				Proximate Analysis (wt%, on Air Dry Basis)				*HHV* (MJ/kg)
	C	H	N	O	*Moisture*	*Ash*	*VM*	*FC*	
D90&SH10	31.91 ± 0.2	5.03 ± 0.07	3.05 ± 0.1	14.61 ± 0.01	4.4 ± 0.02	45.4 ± 0.1	47.1 ± 0.2	3.1 ± 0.09	14.6
D50&SH50	38.85 ± 0.3	5.90 ± 0.05	2.95 ± 0.05	25.90 ± 0.05	4.1 ± 0.05	26.4 ± 0.08	61.4 ± 0.06	8.1 ± 0.04	17.2
D10&SH90	45.09 ± 0.2	6.70 ± 0.03	2.83 ± 0.07	36.18 ± 0.09	4.3 ± 0.01	9.2 ± 0.01	73.1 ± 0.3	13.4 ± 0.1	19.7

**Table 2:** Main stages of thermal decomposition.

Sample	Heating Rate (°C/min)	Temperature Range (*T*_peak_) (°C)			Residual Mass (wt%)
		Dehydration	Devolatilization	Degradation of Char and Minerals	
D90&SH10	5 10 20 30	28–169 (88.9) 25–180 (102.3) 26–182 (107.3) 26–190 (111.8)	165–550 (299.3) 180–548 (307.4) 182–540 (329) 190–530 (314.1)	550–700 (665.8) 548–700 (687.3) 540–700 (686.7) 530–700 (no peak)	58.0 59.0 59.3 56.9
D50&SH50	5 10 20 30	23–175 (86.1) 24–180 (93.8) 23–185 (107.6) 24–200 (128.8)	175–550 (310.4) 180–540 (323.2) 185–525 (330.8) 200–521 (327.3)	550–700 (no peak) 540–700 (no peak) 525–700 (no peak) 521–700 (no peak)	47.1 50.8 40.3 45.2
D10&SH90	5 10 20 30	28–160 (82.3) 25–170 (89.5) 30–186 (101.9) 32–187 (111.1)	160–550 (321.1) 170–530 (325.2) 186–525 (333.2) 187–522 (328.1)	550–700 (no peak) 530–700 (no peak) 525–700 (no peak) 522–700 (no peak)	34.9 33.9 35.4 34.2

**Table 3:** Values of activation energy according to different methods.

α	OFW			KAS			Friedman		
	*E_α_* (kJ/mol)	R^2^	*A* (1/min)	*E_α_* (kJ/mol)	R^2^	*A* (1/min)	*E_α_* (kJ/mol)	R^2^	*A* (1/min)
	D90&SH10								
0.1	232.77	0.96	7.20 × 10^20^	173.14	0.96	1.75 × 10^15^	381.29	0.98	2.70 × 10^34^
0.2	378.42	0.98	1.48 × 10^34^	284.30	0.97	1.98 × 10^25^	393.35	0.97	3.39 × 10^35^
0.3	385.43	0.98	6.43 × 10^34^	289.25	0.99	6.52 × 10^25^	417.27	0.97	5.09 × 10^37^
0.4	414.88	0.94	3.08 × 10^37^	311.70	0.93	8.36 × 10^27^	474.25	0.93	7.72 × 10^42^
0.5	406.74	0.93	5.60 × 10^36^	305.11	0.92	2.88 × 10^27^	517.76	0.88	6.92 × 10^46^
0.6	352.51	0.96	6.44 × 10^37^	262.52	0.83	4.36 × 10^23^	450.19	0.92	5.03 × 10^40^
0.7	400.52	0.95	2.71 × 10^38^	298.67	0.80	6.19 × 10^28^	487.00	0.92	5.55 × 10^46^
Average	367.32		4.40 × 10^37^	274.95		1.05 × 10^28^	445.87		1.78 × 10^46^
	D50&SH50								
0.1	223.45	0.96	2.79 × 10^19^	164.94	0.85	1.55 × 10^14^	299.66	0.99	1.77 × 10^26^
0.2	282.16	0.95	4.88 × 10^24^	209.67	0.96	1.63 × 10^18^	326.25	0.90	4.10 × 10^28^
0.3	305.67	0.94	6.05 × 10^26^	227.50	0.93	6.44 × 10^19^	357.85	0.90	2.63 × 10^31^
0.4	347.37	0.96	3.09 × 10^30^	259.50	0.96	4.66 × 10^22^	413.16	0.91	2.12 × 10^36^
0.5	392.24	0.96	2.96 × 10^34^	293.99	0.99	5.53 × 10^25^	464.18	0.91	6.99 × 10^40^
0.6	280.84	0.97	3.72 × 10^24^	207.59	0.99	1.06 × 10^18^	331.64	0.95	1.24 × 10^29^
0.7	416.89	0.88	4.54 × 10^36^	312.41	0.93	2.41 × 10^27^	579.15	0.86	1.02 × 10^51^
Average	321.23		6.52 × 10^35^	239.37		3.52 × 10^26^	395.99		1.46 × 10^50^
	D10&SH90								
0.1	422.58	0.99	1.23 × 10^37^	318.98	0.89	8.16 × 10^27^	458.32	0.99	1.77 × 10^40^
0.2	334.56	0.96	1.97 × 10^29^	250.25	0.92	6.29 × 10^21^	370.71	0.96	3.15 × 10^32^
0.3	311.83	0.93	1.89 × 10^27^	232.32	0.95	1.58 × 10^20^	347.01	0.92	2.50 × 10^30^
0.4	308.84	0.94	1.03 × 10^27^	229.75	0.96	9.33 × 10^19^	352.46	0.92	7.61 × 10^30^
0.5	323.90	0.92	2.23 × 10^28^	241.18	0.98	9.77 × 10^20^	338.64	0.90	4.53 × 10^29^
0.6	317.04	0.93	5.49 × 10^27^	235.67	0.99	3.15 × 10^20^	312.38	0.89	2.12 × 10^27^
0.7	280.90	0.98	3.38 × 10^24^	207.49	0.99	9.55 × 10^17^	328.56	0.95	5.78 × 10^28^
Average	328.52		1.76 × 10^36^	245.09		1.17 × 10^27^	358.29		2.53 × 10^39^

**Table 4:** Results of the proximate analysis and mineral compositions of pyrolysis char.

	PC550-1	PC550-2	PC550-3
*VM*, wt%	7.60 ± 0.2	16.13 ± 0.7	22.4 ± 0.3
*Ash*, wt%	55.65 ± 0.2	41.05 ± 0.2	31.52 ± 0.1
*FC* *, wt%	36.75 ± 0.3	42.82 ± 0.4	46.08 ± 0.4
Ca **	0.674	1.923	2.525
K **	1.182	2.204	2.632
Zn **	0.008	0.057	0.181
P **	0.094	0.63	0.93
S **	0.034	0.078	0.105
Si **	0.269	2.80	2.678
Al **	0.141	-	0.203
Fe **	0.194	1.10	1.323
Mn **	0.008	0.039	0.053
Br **	0.002	0.004	0.006
Sr **	0.006	0.019	0.021
Zr **	-	0.013	0.017
As **	-	0.002	-
Cr **	0.03	0.005	0.007
Rb **	0.003	0.006	0.008

* *FC* = 100 − *VM* − *Ash*; ** Mass fraction of major elements in original ashes, rel%.

**Table 5:** IR spectroscopic analysis of pyrolysis char.

Wavenumber, cm^−1^	Stretching Vibrations	Samples
3000–3600	O–H bonds (in alcohols, phenols, and residual water)	PC550-1, PC550-3
1587–1594	C=C bonds in aromatic rings	PC550-1, PC550-3
1410–1411; 874	CaCO_3_ bonds	PC550-1, PC550-2, PC550-3
1300–1150	P=O bonds	PC550-1, PC550-3
1080–1100	Si–O–Si bonds in silicon dioxide	PC550-1, PC550-2, PC550-3
1050–1150	C–O–C and C–O bonds in ethers and alcohols	PC550-1, PC550-3
754–772	C–H bonds	PC550-1, PC550-2, PC550-3

**Table 6:** Chemical composition of pyrolysis liquid.

Group	Subgroup	PL550-1	PL550-2	PL550-3
Hydrocarbons	Saturated hydrocarbons	10.43	2.62	5.02
	Unsaturated hydrocarbons	2.1	0.06	0.14
	Cyclic hydrocarbons	2.51	0.68	-
	Aromatic hydrocarbons	5.62	1.61	2.82
Oxygen-containing compounds	Carboxylic acids	4.56	17.98	26.73
	Carboxylic acid esters	4.31	5.95	6.73
	Alcohols	0.55	5.63	3.36
	Aldehydes	0	0.26	2.21
	Ketones	3.45	11.08	6.79
	Phenol	14.89	19.07	20.39
	Heterocyclic compounds	0.79	3.74	1.24
Nitrogen-containing compounds	Amides	1.27	0.81	-
	Nitriles	1.49	1.09	-
	Heterocyclic compounds	4.77	1.47	-
	Other	43.26	27.95	24.57

**Table 7:** Comparative analysis of kinetic and thermodynamic parameters.

Materials	*E_α_* (kJ/mol)	ΔG (kJ/mol)	ΔH (kJ/mol)	ΔS (J/mol∙K)	Reference
SH	79.11–162.57	R1: 147.89 R2: 169.86 R3: 170.72	R1: 86.21 R2: 165.24 R3: 67.66	R1: −120.94 R2: −7.82 R3: −179.24	[66]
Digestate with lignocellulosic biomass (*Typha latifolia*)	62–401	161–170	65–397	(−180)–(406)	[67]
Chicken manure	108.1	-	-	-	[29]
Chicken manure	167.5–213.9	157.01	162.52		[46]
				91.77	
Chicken manure	150–320	130–210	135–320	0–150	[43]
Chicken manure	149–288	158–175	165–170	(−8)–(12)	[68]
Canola residue	129–391	158–212	136–385	(−51)–(284)	[69]
Sugarcane bagasse (BG)	110–180.9	-	-	-	[27]
Rice straw (RS)	110–180.9	-	-	-	[27]
Rice husk (RH)	124–195	-	-	-	[27]
Chicken manure	140.4–151.2	239.8–245.3	134.1–145	(−150)–(−130)	[48]
Wheat straw	65.2 (OFW) 63.4 (KAS)	170–184 (OFW) 176–190 (KAS)	62 (OFW, KAS)	(−192)–(−169) (OFW) (−212)–(−188) (KAS)	[26]
Peel of karanja (*Pongamia pinnata*) fruit	68.53 (FWO) 62.13 (KAS)	-	-	-	[49]
Tea Factory Waste (TFW)	199.5046 (KAS) 199.1154 (OFW) 207.9710 (Friedman)	152.33 (KAS) 152.09 (OFW) 158.17 (Friedman)	194.58 (KAS) 194.20 (OFW) 203.05 (Friedman)	70 (KAS) 70 (OFW) 80 (Friedman)	[70]
Pistachio shell	128.94–179.08	171.85	164.17–174.10	(−235.28)–(−101.02) (FWO) (−243.19)–(−103.79) (KAS) (−231.13)–(121.92) (Friedman)	[71]
Scots pine (*Pinus sylvestris*) needles	156.89	159.99	151.92	−13.51	[57]
RH	219.67 (FWO) 222.18 (KAS)	173.30–178.88 (FWO) 173.25–178.82 (KAS)	214.60 (FWO) 217.11 (KAS)	57.24–66.38 (FWO)	[58]
Corn stalk	206.2 (KAS)	196.79 (KAS)	200.67 (KAS)	(−29)–(93.82) (KAS)	[72]
Ash sawdust	198 (Friedman)	181.71–185.48 (Friedman)	174.74–352.57 (Friedman)	(−16.64)–(267.80) (Friedman)	[73]
Wood chips	144–433	-	-	-	[74]
Bedding material	99–484	-	-	-	[74]
Broiler waste	99–464	-	-	-	[74]
Sunflower stalks	207.08–505.54 (FWO) 208.89–518.4 (KAS)	158.98–164.99	126.83–413.11	(−61.22)–407.83	[75]
Chicken litter	100–470	-	-	-	[76]
Poultry litter	200–250	-	-	-	[77]
Chicken litter	242.3	-	-	-	[78]
D90&SH10	232.77–414.88 (OFW) 173.14–311.70 (KAS) 381.29–517.76 (Friedman)	143.28–146.29 (OFW) 144.72–147.74 (KAS) 142. 92–143.83 (Friedman)	227.94–420.43 (OFW) 166.99–314.61 (KAS) 412.44–512.93 (Friedman)	140.52–458.9 (OFW) 33.04–275.79 (KAS) 400.4–637.96 (Friedman)	This study
D50&SH50	223.45–416.89 (OFW) 164.94–312.41 (KAS) 299.66–579.15 (Friedman)	147.69–150.88 (OFW) 149.17–152.42 (KAS) 145.97–148.95 (Friedman)	218.49–387.28 (OFW) 159.98–307.45 (KAS) 294.70–574.19 (Friedman)	113.28–442.78 (OFW) 12.66–265.21 (KAS) 243.49–522.96 (Friedman)	This study
D10&SH90	280.90–422.58 (OFW) 207.49–318.98 (KAS) 312.38–458.32 (Friedman)	147.92–149.26 (OFW) 149.38–152.05 (KAS) 147.5–149.5 (Friedman)	275.93–417.61 (OFW) 202.52–314.01 (KAS) 307.41–365.74 (Friedman)	210.57–451.05 (OFW) 123.3–275.34 (KAS) 264.12–511.53 (Friedman)	This study

## Data Availability

The original contributions presented in this study are included in the article. Further inquiries can be directed to the corresponding author.

## Appendix A

The dependencies of ln *A* on *E*_α_ were plotted, and the linear correlation of the obtained results was verified.

**Table A1:** Mean *E*_α_ (kJ/mol) ± SD.

Samples	OFW	KAS	Friedman
D90&SH10	367 ± 63	275 ± 48	446 ± 51
D50&SH50	321 ± 68	239 ± 52	396 ± 99
D10&SH90	329 ± 45	245 ± 35	358 ± 48

The relationship between the l ln*A* and *E*_α_ values calculated for the samples under study is shown in Figure A1. As can be seen from the figure, all samples exhibit good linear correlations between ln*A* and *E*_α_ (R^2^ = 0.97–1).
